# Circular RNAs and Their Linear Transcripts as Diagnostic and Prognostic Tissue Biomarkers in Prostate Cancer after Prostatectomy in Combination with Clinicopathological Factors

**DOI:** 10.3390/ijms21217812

**Published:** 2020-10-22

**Authors:** Hannah Rochow, Monika Jung, Sabine Weickmann, Bernhard Ralla, Carsten Stephan, Sefer Elezkurtaj, Ergin Kilic, Zhongwei Zhao, Klaus Jung, Annika Fendler, Antonia Franz

**Affiliations:** 1 Department of Urology, Charité-Universitätsmedizin Berlin, 10117 Berlin, Germany; hannah.rochow@charite.de (H.R.); mchjung94@gmail.com (M.J.); sabine.weickmann@charite.de (S.W.); bernhard.ralla@charite.de (B.R.); carsten.stephan@charite.de (C.S.); zhaozhongweixy@163.com (Z.Z.); annika.fendler@crick.ac.uk (A.F.); antonia.franz@charite.de (A.F.); 2 Berlin Institute for Urologic Research, 10115 Berlin, Germany; 3 Institute of Pathology, Charité-Universitätsmedizin Berlin, 10117 Berlin, Germany; sefer.elezkurtaj@charite.de (S.E.); e.kilic@pathologie-leverkusen.de (E.K.); 4 Institute of Pathology, Hospital Leverkusen, 51375 Leverkusen, Germany; 5 Department of Urology, Qilu Hospital of Shandong University, Jinan 250012, China; 6 Max Delbrueck Center for Molecular Medicine in the Helmholtz Association, Cancer Research Program, 13125 Berlin, Germany; 7 Cancer Dynamics Laboratory, The Francis Crick Institute, 1 Midland Road, London NW1 1AT, UK

**Keywords:** prostate cancer, microarray, identification, validation and differential expression of circular RNAs, circular RNAs and linear counterparts, biochemical recurrence, diagnostic and prognostic tissue biomarkers, improved predictive accuracy by RNA signature

## Abstract

As new biomarkers, circular RNAs (circRNAs) have been largely unexplored in prostate cancer (PCa). Using an integrative approach, we aimed to evaluate the potential of circRNAs and their linear transcripts (linRNAs) to act as (i) diagnostic biomarkers for differentiation between normal and tumor tissue and (ii) prognostic biomarkers for the prediction of biochemical recurrence (BCR) after radical prostatectomy. In a first step, eight circRNAs (circ*ATXN10*, circ*CRIM1*, circ*CSNK1G3*, circ*GUCY1A2*, circ*LPP*, circ*NEAT1*, circ*RHOBTB3*, and circ*STIL*) were identified as differentially expressed via a genome-wide circRNA-based microarray analysis of six PCa samples. Additional bioinformatics and literature data were applied for this selection process. In total, 115 malignant PCa and 79 adjacent normal tissue samples were examined using robust RT-qPCR assays specifically established for the circRNAs and their linear counterparts. Their diagnostic and prognostic potential was evaluated using receiver operating characteristic curves, Cox regressions, decision curve analyses, and C-statistic calculations of prognostic indices. The combination of circ*ATXN10* and lin*STIL* showed a high discriminative ability between malignant and adjacent normal tissue PCa. The combination of lin*GUCY1A2*, lin*NEAT1*, and lin*STIL* proved to be the best predictive RNA-signature for BCR. The combination of this RNA signature with five established reference models based on only clinicopathological factors resulted in an improved predictive accuracy for BCR in these models. This is an encouraging study for PCa to evaluate circRNAs and their linRNAs in an integrative approach, and the results showed their clinical potential in combination with standard clinicopathological variables.

## 1. Introduction

Prostate cancer (PCa) is the second most common cancer type among men [1]. Following radical prostatectomy, which is used as a therapeutic curative option for patients suffering from PCa, biochemical recurrence (BCR), defined as a re-increased serum concentration of prostate-specific antigen (PSA) of >0.2 µg/L [2,3], is clinically considered to be the first sign of disease recurrence [4,5]. An evaluation of six recent studies with more than 1000 patients in each showed that approximately 15–34% of surgically treated patients suffered from BCR within 5 to 10 years after surgery [6]. Following BCR without secondary therapy, distant metastasis manifests in approximately 30% of patients and 19–27% will die within 10 years [7,8]. These data clearly show that early and reliable prediction of patients with a high risk of BCR is necessary to optimize the frequency of follow-up and thus the decision to undergo adjuvant therapy.

For BCR risk prediction, numerous scoring systems based on clinicopathological factors such as the Gleason score or the respective International Society of Urological Pathology (ISUP) grade, pathological tumor stage (pT stage), and preoperative PSA level are currently applied. Among these tools are the Cancer of the Prostate Risk Assessment Postsurgical Score (CAPRAS) [9] and those developed by D’Amico et al. [10], Stephenson et al. [7], and the National Comprehensive Cancer Network (NCCN) [11]. Although all clinicopathological factors are, to some extent, associated with patient outcome, the prognostic accuracy of these nomograms is generally unsatisfactory [12,13,14,15,16]. In this context, prognostic molecular biomarkers could significantly improve the predictive accuracy of tools based only on clinicopathological factors [17,18,19,20,21]. We recently elaborated a five-microRNA signature that outperforms the BCR scoring systems mentioned above [6]. In addition, the combined use of clinicopathological factors and molecular markers was found to significantly improve the predictive accuracy compared to the separately calculated predictive value [6]. Based on this experience using molecular markers in BCR prediction, we decided to extend this approach to circular RNAs (circRNA), which we successfully introduced as prognostic biomarkers in clear cell renal cell carcinoma [22].

Recently, circRNAs have the subject of increasing interest in medicine. These RNAs consist of a single strand of RNA in a closed loop [23,24], and are formed by alternative splicing of mostly exonic sequences. One host gene can form several different circRNAs [25,26]. The functions of circRNAs are still being investigated. Their ability to sponge microRNAs (miRNAs), a process in which circRNAs prevent the inhibitory properties of miRNA and therefore promote the expression of target mRNAs, is relatively well known [23,24,27]. Furthermore, circRNAs may regulate the expression of their parenteral genes by interacting with RNA polymerase II [28], but they can also interact with RNA-binding proteins and are therefore involved in the regulation of gene expression [29]. Some circRNAs may even be able to encode proteins [30]. CircRNAs have expression patterns that are specific to different cells or tissues and have been shown to play roles in cell regulation, including physiological as well as pathological processes [24,31,32,33,34,35]. It has been shown that circRNAs can act as oncogenes and tumor suppressors in the initiation and progression of different cancers, e.g., hepatocellular carcinoma, gastric carcinoma, colorectal cancer, and renal cell carcinoma [22,36,37,38,39]. All of these aspects justify a particular interest in using circRNAs as biomarkers in diagnosis, prognosis, and prediction, as well as for therapeutic targets [33,40,41,42,43].

CircRNAs in PCa are also a subject of present research. Last year, Chen et al. [44] identified a broad signature of PCa-specific circRNAs via ultra-deep rRNA-depleted RNA sequencing of localized PCa tissue samples. Moreover, specific circRNA functions were shown. Circ*CSNK1G3*, for example, seems to promote cell proliferation in PCa [44]. Furthermore, Zhang et al. [45] applied a bioinformatics approach using various PCa cells to identify numerous circRNAs, including circ*GUCY1A2*, as potential candidates for PCa progression. Other working groups using microarray platforms have reported lists of the top up- and downregulated circRNAs in PCa tissue compared with adjacent normal tissue [46,47]. Recent studies have particularly analyzed functional features and underlying molecular mechanisms of individual circRNAs [48,49,50,51,52,53]. Several publications particularly focused on androgen receptor pathway related circRNAs [54,55,56,57]. Correlations between the expression of circRNAs and relevant clinicopathological factors or survival Kaplan-Meier analyses have been reported [44,51,58,59,60,61,62]. However, it is astonishing that the potential of circRNAs as diagnostic and prognostic tissue biomarkers has so far only been evaluated in isolated cases [47,58,61]. As far as we know, only one study has ever conducted multivariate analyses of circRNAs in connection with clinicopathological factors [61]. Studies on the clinical validity of circRNAs in relation to BCR are still lacking.

Thus, in this study, we aimed to (i) identify differentially expressed circRNAs in six paired samples of PCa tissue and adjacent normal tissue using microarray analysis, (ii) validate the differential expression of eight chosen circRNAs and their linear counterparts via reverse-transcription quantitative real-time polymerase chain reaction (RT-qPCR), (iii) examine the differentiating potential between malignant and non-malignant prostate tissue in 194 samples of 115 PCa patients including 79 paired samples, and (iv) evaluate the potential of the chosen circRNAs and their linear counterparts as biomarkers in combination with the clinicopathological factors of PCa patients after radical prostatectomy to predict BCR.

## 2. Results

### 2.1. Patient Characteristics and Study Design

**Figure 1:**
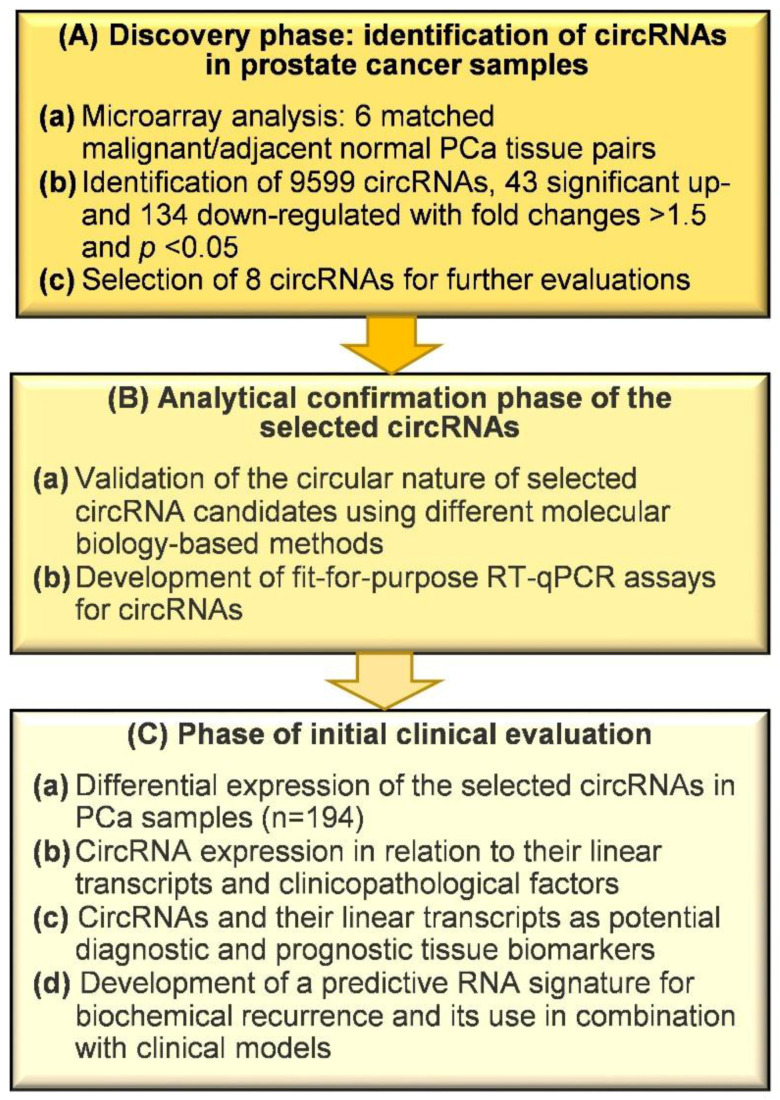
Workflow of the study in three phases. Abbreviations: circRNA, circular RNA; PCa, prostate cancer; RT-qPCR, reverse-transcription quantitative real-time polymerase chain reaction.

One hundred and fifteen untreated PCa patients who underwent radical prostatectomy between 2007 and 2014 with follow-up data until November 2019 were included in this study. Follow-up data were based on medical records and telephone contacts with the patients, their physicians, and their family members. In total, 194 tissue samples with 79 pairs of adjacent normal and malignant samples, and 36 with malignant characteristics only were investigated (Table 1). The sample size was determined using a power-adapted calculation (α = 5%, power = 80%; Supplementary Information S1 (Supplementary Materials)). A two-to-one selection of available samples, based on patients with BCR, was retrospectively performed with 76 patients without BCR and 39 with BCR. BCR was defined as a postoperative PSA increase above 0.2 µg/L after radical prostatectomy, as confirmed by consecutive increased values [2,3]. The workflow diagram presented in Figure 1 outlines the design of this study, which involved three phases based on the above postulated objectives for this investigation: (i) the discovery phase of identifying differentially expressed circRNAs using a microarray screening approach and the selection of circRNAs for further evaluation; (ii) analytical confirmation of the circular nature of selected circRNAs and elaboration of “fit-for-purpose” RT-qPCR assays for circRNAs and their linear transcripts; and (iii) initial clinical evaluations regarding their validity as discriminative tissue classifiers and the predictive value of these biomarkers when applied alone and in combination with conventional clinicopathological factors.

### 2.2. Discovery of circRNAs in Prostate Cancer Tissue Using Microarray Analysis

#### 2.2.1. Identification of Differentially Expressed circRNAs

**Figure 2:**
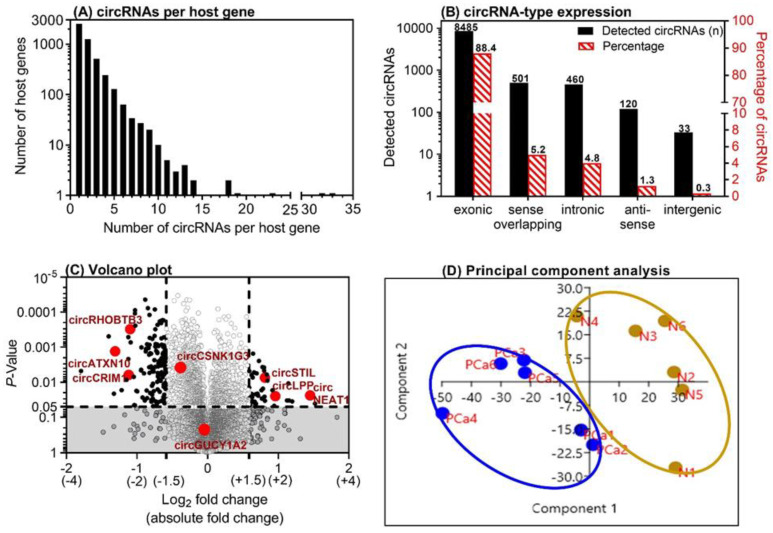
Microarray analysis results of six matched prostate cancer (PCa) tissue samples. (**A**) The number of circular RNAs (circRNAs) expressed per host gene in the malignant tissue samples and their matched adjacent normal tissue samples from PCa specimens after prostatectomy. (**B**) Genomic origin of the detected circRNAs on the microarray. (**C**) Volcano plot with the up- and downregulated circRNAs in malignant vs. adjacent normal tissue samples. The dashed lines indicate the thresholds: absolute 1.5-fold changes and *p*-values of 0.05 in the *t*-test. The eight circRNAs that were selected for further evaluation in this study are marked. (**D**) Results of the principal component analysis with the left cluster of tumor samples (PCa1–PCa6, marked in blue) and the right cluster with the paired adjacent normal tissue samples (N1–N6, marked in brown).

Six matched PCa tissue samples were examined using ArrayStar microarray experiment. A total of 9599 circRNAs out of 13,617 distinct probes on the array were detected (Supplementary Microarray Data File.xlsx (Supplementary Materials)). This number of circRNAs derived from 4838 host genes, since numerous host genes can form multiple circRNA isoforms [26]. Approximately 26% of all detected circRNAs were found to be from ~50% of the host genes that form only one circRNA, while the other 41% of circRNAs were found from the ~35% of the host genes that can form two or three circRNAs. Approximately 3.2% of the circRNAs were derived from only 0.6% of the host genes able to form 10 or more circRNAs (Figure 2A). Different genomic regions can be the origin of circRNAs. In this PCa microarray screening, approximately 90% of circRNAs were of exonic origin, but intronic, sense-overlapping, anti-sense, and intergenic circRNA types were also detected (Figure 2B). These data correspond with our own results in renal cell carcinoma [22] and data from studies on other tissues [25]. Regarding the differential expression between adjacent normal and malignant tissue, the array data identified 43 upregulated and 134 downregulated circRNAs with a higher than absolute 1.5-fold change (*p* < 0.05) in malignant tissue samples (Figure 2C). Using a threshold of 2-fold change, only six upregulated and 18 downregulated circRNAs were identified. Based on a principal component analysis of the microarray expression data, two separate clusters with malignant and adjacent normal tissue characteristics were ascertained (Figure 2D).

#### 2.2.2. Selection of circRNAs for Further Evaluation

In addition to the microarray-based expression results (absolute fold-change >1.5 with *unadjusted* *p* < 0.05 and sufficiently raw intensity on the microarray), we used interest-specific criteria to select circRNAs for further investigation. We selected six circRNAs (Table 2), for which no information on prostate carcinoma was available. Their host genes had been described in individual studies with regard to their roles in either PCa progression (e.g., *CRIM1*, *NEAT1*, and *STIL* [63,64,65]) or other cancers (e.g., *LPP* and *RHOBTB3* [66,67]). Some of the selected circRNAs had been partly identified in other cancers (e.g., circ*CRIM1* and circ*RHOBTB3* [22,68]). Finally, an in silico analysis of miRNA interaction with these circRNAs was performed using the algorithm provided by the CircInteractome tool and the miRDB and TargetScan databases [69,70,71]. In all cases, the circRNAs were found to be crucial points for potentially relevant miRNA–gene interactions (Figure S1). This also fulfilled a selection criterion for further investigations to be planned. *NEAT1* was identified as a special case because it already has miRNA-sponging functions as a long non-coding RNA (lncRNA) [65]. Thus, the relationship between the circRNA and the lncRNA transcript was of particular interest. In this circRNA panel, two additional circRNAs from the genes *CSNK1G3* and *GUCY1A2* were included as these circRNAs were recently identified in PCa tissue samples and PCa cell lines as mentioned in the introduction [44,45]. Collectively, the microarray analysis of the six paired samples in the discovery phase (Figure 1) must be considered an exploratory study for ranking deregulated circRNAs using the unadjusted *p*-values supported by the mentioned additional selection criteria. Under these conditions, an exploratory study should be preferably analyzed without *p*-value adjustment, but needs a technical replication by a different assay technique and biological validation using other clinical samples [72,73,74]. This was done in the two subsequent workflow phases B and C (Figure 1).

Data on the selected circRNAs are given in Table 2. Currently, there is no standardized nomenclature for circRNAs [22], and the designations of the database circBase are mainly used as a reference [75]. In order to facilitate the readability of the manuscript, official gene symbols with the prefixes “circ” and “lin” are used herein to characterize our selected circRNAs and the corresponding linear transcripts from the same host gene (Table 2).

### 2.3. Analytical Confirmation Phase of the Selected circRNAs

#### 2.3.1. Experimental Proof of the Circular Nature of Transcripts

**Figure 3:**
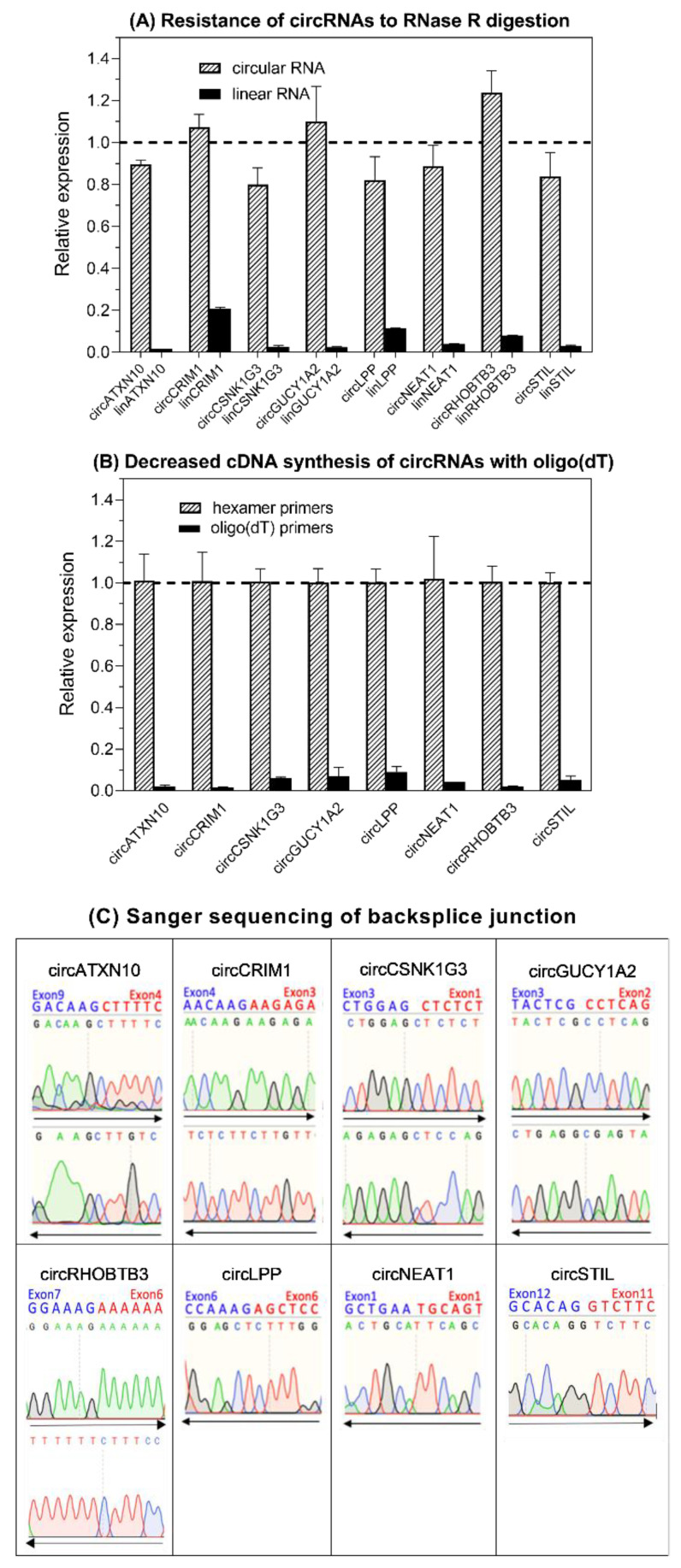
Experimental proof of the circular nature of the circRNAs selected in this study. (**A**) Resistance of circRNAs to RNase R digestion compared with linear RNAs. Data for triplicates (mean ± standard deviation) normalized to controls without RNase treatment are presented. (**B**) Decreased cDNA synthesis of circRNAs with oligo(dT)_18_ vs. random hexamer primers. Data are given as the relative expression normalized to hexamer-primers-based cDNA synthesis. The relative expression was markedly decreased in all circRNAs (at least *n* = 3 of tissue pools) when using oligo(dT)_18_ primers in comparison to random hexamer primers, indicating that the circRNAs lacked a poly(A) tail. (**C**) Base sequence of circRNA backsplice junction pictured by Sanger sequencing. Circ*LPP*, circ*NEAT1*, and circ*STIL* were only sequenced in one direction as one of the primers was junction-spanning (Table S3). The sequencing result of circ*RHOBTB3* corresponded to that in kidney carcinoma [22]. Methodical details for all experiments listed here are described in Section 4 and Supplementary Information S4 (Supplementary Materials).

RT-qPCR assays using SYBRGreen I were established for the eight selected circRNAs and their linear counterparts, taking into account the MIQE guidelines “Minimum Information for Publication of Quantitative Real-Time PCR Experiments” [76] (Supplementary Information S3 (Supplementary Materials) with the Tables S1–S7 in addition to Section 4 of this paper). Experimental confirmation of the circular nature of the identified circRNAs via microarray and sequencing technologies was achieved using different tests to confirm the characteristics of the circRNA-specific backsplice junction [24,77,78]. Figure 3 shows that circRNAs are resitant to RNase R digestion (Figure 3A), distinctly decreased complementary DNA (cDNA) synthesis occurred when oligo(dT)_18_ primers were used compared with when random hexamer primers (Figure 3B) were used, and the backsplice junction was confirmed by Sanger sequencing (Figure 3C). Melting curve analysis and electrophoresis of the amplicons were applied in order to validate the analytical specificity of the RT-qPCR products in all assays (Figures S2, S3, and Table S7).

#### 2.3.2. Analytical Performance of RT-qPCR Assays

According to the MIQE guidelines [76] and also to the “Standards for Reporting of Diagnostic Accuracy Studies” (STARD) [79], the repeatability (intra-assay variation) and reproducibility (inter-assay variation) of the measurements should be used decisive criteria for the performance and robustness of RT-qPCR tests. Supported by the analytical specificity of the established assays (Figures S2 and S3) and the characteristics of the PCR standard curves (Table S7), the data shown in Table 3 proved that the assays were suitable for “fit-for-purpose” RT-qPCR measurements in clinical studies [80]. An exception was circ*NEAT1*, which had rather poor repeatability and reproducibility due to its very low expression.

### 2.4. Clinical Assessment

#### 2.4.1. Differential Expression of circRNAs in Relation to Clinicopathological Variables

In the first step, we compared the circRNA expression data obtained from the six paired tumor and adjacent normal tissue samples using microarray analysis and the established RT-qPCR assays (Table 4). The expression results were in good agreement between both measurement methods for the circRNAs, with the exceptions of circ*LPP* and circ*STIL*. Circ*LPP* and circ*STIL* were found to be upregulated in the microarray analysis but downregulated in RT-qPCR measurements. Despite this clear discrepancy, we decided to include these two circRNAs together with their linear transcripts in further analyses.

**Figure 4:**
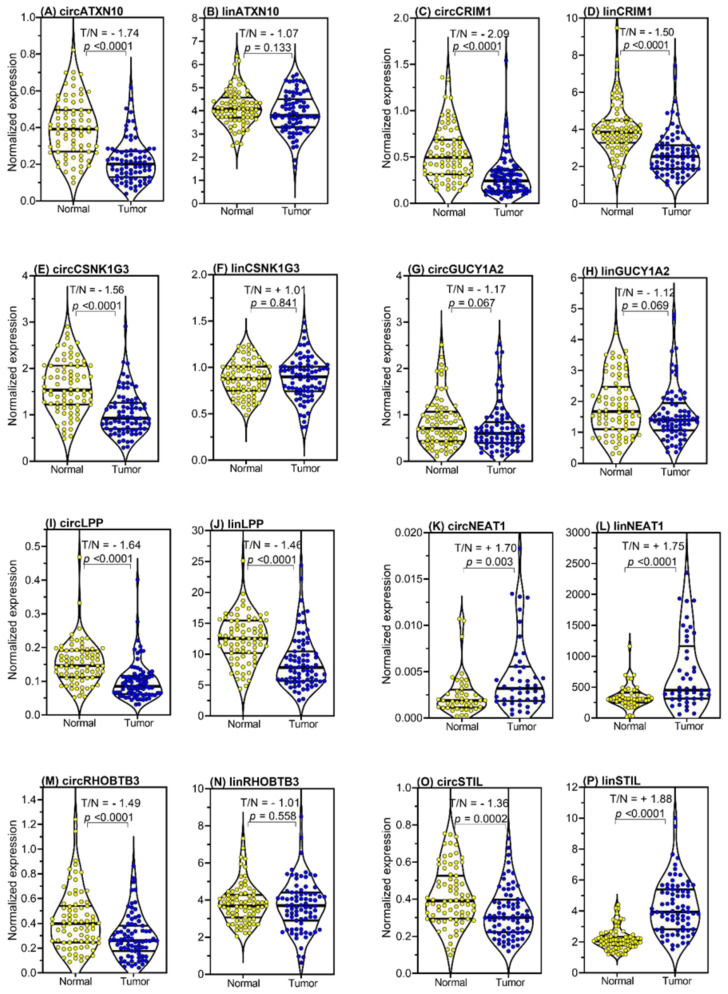
Expression levels of circular RNAs (circRNAs) and the linear transcripts of their host genes in tissue samples from prostate cancer (PCa) patients. The expression data of all eight circRNAs (**A**,**C**,**E**,**G**,**I**,**K**,**M**,**O**) and their corresponding linear transcripts (**B**,**D**,**F**,**H**,**J**,**L**,**N**,**P**) are shown in the matched pairs of adjacent normal tissue samples and malignant samples from PCa specimens collected by radical prostatectomy (*n* = 79, only 45 for circ*NEAT1* and lin*NEAT1*). *ALAS1* (5′-aminolevulinate synthase 1) and *HPRT1* (hypoxanthine phosphoribosyltransferase 1) mRNAs were used as stable expression normalizers of prostatic cancer [81]. Complete violin plots with the entire expression ranges, the lower and upper quartiles (dashed lines), and the medians (bold lines) are presented. Statistically significant expression differences of the malignant tissue samples compared with the adjacent normal tissue samples are given as the T/N (tumor/normal) index. To facilitate a direct comparison of the expression results of each circRNA and its corresponding linear transcript in the tumor to normal tissue, we used the term T/N index. A positive number indicates a higher expression in tumor tissue (numerator in the index) in relation to normal tissue (denominator in the index) and a negative number shows a higher expression in the normal tissue (denominator in the index) in relation to tumor tissue (numerator in the index).

The expression levels of the circRNAs and their linear transcripts were measured and evaluated in all samples of the studied cohort (*n* = 194). To examine the differential expression between adjacent normal and malignant tissue samples, only the expression results of the paired samples of adjacent normal and malignant tissue samples were compared, in order to avoid bias due to biological variations between patients (Figure 4). Significant differential expression was observed between tumor and normal tissue samples, as indicated by T/N indices (Figure 4), for both circRNAs and their corresponding linear transcripts (Figure 4 and detailed in Table S8). The expression levels of all circRNAs except circ*NEAT1* and circ*GUCY1A2* were downregulated in tumor samples; circ*NEAT1* was upregulated and the expression level of circ*GUCY1A2* did not differ between the two tissue samples. In contrast, only the linear transcripts lin*CRIM1* and lin*LPP* were downregulated in the tumor samples, while lin*STIL* and lin*NEAT1* were upregulated, and lin*ATXN10*, lin*CSNK1G3*, lin*GUCY1A2*, and lin*RHOBTB3* showed no significant differences in expression between the normal and tumor tissue samples.

Moreover, the following characteristics of the expression data were striking: (i) the upregulation of circLPP and circ*STIL* found in the microarray analysis could not be confirmed, as both circRNAs were shown to be downregulated in the RT-qPCR measurements, similarly to what was shown by the comparison of the paired samples used in the microarray analysis; (ii) all linear transcripts except circ*CSNK1G3* had significantly higher expression than their circRNAs. This can be seen on the x-axes of the corresponding panels and by the different ratios between the two tissue samples and the RNA types, as summarized in Tables S9 and S10. This was particularly remarkable for circ*NEAT1*, which showed a low expression rate, with only 45 of the 79 examined pairs having detectable expression. Although the high number of biological replicates confirmed the increased expression of this circRNA in tumor tissue, circ*NEAT1* was not included in the further multivariable prognostic BCR analysis. This was also compatible with the less reliable analytical performance data of circ*NEAT1* measurements in its low expression range, as mentioned above (Table 3), and the number of samples above the upper limit of the standard curve of this circRNA (Table S7).

The expression data of all examined circRNAs and their linear transcripts in the tumor samples were not associated with age, preoperative PSA, prostate volume, digital rectal examination, tumor stage, and surgical margin status (Table S11). However, significant associations between the ISUP grade and all circRNAs except circ*NEAT1* and circ*RHOBTB3* were found, while only lin*GUCY1A2* and lin*LPP* showed such associations for the linRNAs.

Close correlations between the expression levels of all circRNAs in both the adjacent normal and malignant tissue samples were observed, except for circ*NEAT1* and partly for circ*RHOBTB3* (Tables S12 and S13). However, these close correlations were mainly lost if data in matched normal tissue and malignant tissue samples were correlated (Table S14). Furthermore, there were several different correlation coefficients between circRNAs and the linear transcripts in malignant tissue samples in comparison to the matched adjacent normal tissue samples (Table S15).

#### 2.4.2. CircRNAs and linRNAs as Biomarkers for Discrimination between Normal and Cancerous Tissue

**Figure 5:**
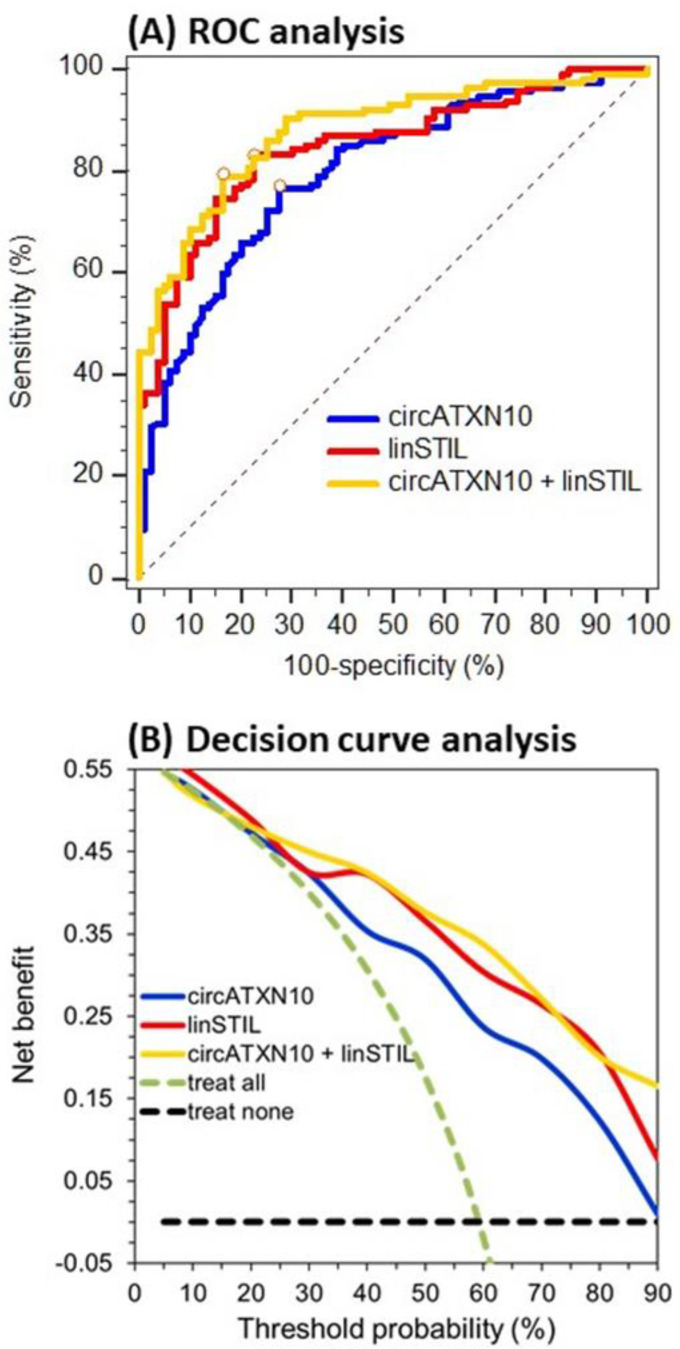
Receiver operating characteristic (ROC) curve and decision curve analyses of circ*ATXN10* and lin*STIL* as individual markers and in combination for discrimination between adjacent normal and malignant tissue samples. The data reflect the results shown in Table 5 for circ*ATXN10*, lin*STIL*, and their combination.

The differences between the circRNAs and their linear transcripts described here support the idea, postulated in the introduction, that it makes sense to investigate circRNAs as cancer biomarkers in an integrative approach together with their linear transcripts, due to their potential differential influences in normal and cancerous tissue. From this point of view, the expression data of the circRNAs and linear transcripts were used to differentiate between adjacent normal and malignant tissue (Table 5). Data from the performed receiver operating characteristic (ROC) curve analysis revealed that circ*ATXN10* and lin*STIL* were found to be the best individual markers for this purpose, with areas under the curves (AUCs) of 0.801 and 0.841, respectively. Using a backward elimination approach of binary logistic regression with all RNAs, a combined tool using these two markers resulted (Table 5). It is of particular interest that both RNAs were differentially expressed. Circ*ATXN10* was downregulated in tumor samples, whereas lin*STIL* was upregulated. When applying the markers combined, the AUC value increased to 0.892. Both the ROC curve and the decision curve of this combination were found to run above the curves of the two individual markers (Figure 5). Thus, at least a “stabilizing” discriminative ability was achieved with the marker combination of circ*ATXN10* + lin*STIL*.

#### 2.4.3. CircRNAs and Linear Transcripts as Potential Markers for Predicting BCR

BCR, as the selected clinical outcome endpoint, was defined as the time from the radical prostatectomy until the time of the corresponding event or the last follow-up. Detailed data for the patients with and without BCR at the time of follow-up after surgery are shown in Table 1.

According to the Reporting Recommendations for Tumor Marker Prognostic Studies (REMARK) [82], we used continuous data of the normalized relative expression quantities of the RNAs in the subsequently described Cox regression analyses. This procedure of using continuous data, if possible, is strongly recommended to avoid loss of information in detecting associations between cancer markers and time-dependent events [82]. The results of the univariable Cox regression analyses in this first step, which was done to evaluate the potential predictive validity of the total RNA panel, are shown in Table 6. Those five circRNAs and three linear transcripts with *p*-values < 0.25 were selected for subsequent multivariable Cox regression analyses to avoid type II errors. So-called “full models”, including the respective circular and linear RNAs, and “reduced models” after a backward elimination (entry: *p* < 0.05, removal: *p* > 0.100) were separately constructed for the circRNAs and linRNAs (Table 6).

For the circRNAs, only circ*ATXN10*, circ*GUCY1A2*, and circ*LPP* remained in the reduced model, while for the linRNA-based model (lin*GUCY1A2*, lin*NEAT1*, and lin*STIL*), no further variables were eliminated by the backward approach. To estimate the capacity of these models to predict BCR, the C-statistic values were compared. The C-statistic results, given as the AUC ± SE of the prognostic indices calculated in the Cox regression analyses, did not differ between the full and reduced models for the circRNA-based BCR prediction (0.676 ± 0.055 vs. 0.649 ± 0.056, *p* = 0.219; details given in Table S16). The linRNA-based C-statistic value was found to be 0.722 ± 0.053, but it was also not statistically significant compared with the circRNA-based model (*p* = 0.141; details given in Table S16). However, a Cox regression analysis with eight RNA variables from the circRNA-based and linRNA-based “full models” or the six RNAs of the “reduced models” (Table 6) and a subsequent backward elimination showed that all circRNAs were excluded from the model (Table 7). Only three linRNAs—lin*GUCY1A2*, lin*NEAT1*, and lin*STIL*—remained as independent variables in the model. This clearly shows that, compared to these linear RNAs, the circRNAs did not contribute to the BCR prediction. Thus, the model with lin*GUCY1A2*, lin*NEAT1*, and lin*STIL*, termed the “RNA signature” in the following text, was used as an additional tool for BCR prediction, together with clinicopathological factors.

To assess the validity of our linear transcript data regarding the BCR prediction, we used The Cancer Genome Atlas Prostate Cancer (TCGA-PRAD) dataset, a publicly available dataset (Table S17). This dataset contains information from 427 patients and includes 89 cases of BCR, defined as a re-increase of PSA > 0.2 µg/L after prostatectomy, as in our study cohort. Univariable Cox regression analyses of the linear transcripts showed that increased expression of lin*STIL* was closely associated with BCR, as in our study, whereas statistically significant relationships of the other transcripts with BCR were not observed (Table S17).

#### 2.4.4. BCR Prediction Models Based on Clinicopathological Variables in Combination with the RNA Signature

As briefly outlined in the introduction, different tools for predicting BCR based on the clinicopathological variables have been introduced in clinical practice. It was therefore of interest to (i) compare the predictive potential of the RNA signature elaborated above with the results of such clinical models and (ii) evaluate whether a combination of both approaches could improve the prognostic accuracy of single tools.

For this purpose, based on univariable and multivariable Cox regression analyses of the clinicopathological variables in our study cohort, we constructed full and reduced models to predict the occurrence of BCR (Table 8).

In addition, the established predictive BCR reference models CAPRAS [9] and NCCN [11] as well as those according to D’Amico et al. [10] and Stephenson et al. [7] were used. In all cases, the C-statistic values of the obtained prognostic indices were calculated using purely clinicopathological-based tools combined with the RNA signature (Table 9). As mentioned above, the C-statistic value of the RNA signature with lin*GUCY1A2*, lin*NEAT1*, and lin*STIL* was 0.722 ± 0.053 (95% CI: 0.631–0.801), which was only significantly higher than D’Amico et al.’s reference model value of 0.513 (95% CI: 0.418–0.607; *p* = 0.003). There were no statistical differences compared with the other clinicopathological tools listed in Table 9 (*p*-values between 0.128 and 0.640). However, the combination of the RNA signature with individual clinicopathological-based prediction tools increased the C-statistics values of all clinicopathological-based tools (Table 9). This was especially statistically significant for the tools presented by D’Amico et al. [10], CAPRAS [9], and NCCN [11]. In addition, decision curve analyses of our elaborated full model and the other four clinicopathological-based reference models were performed in combination with the RNA signature (Figure S4). The improved prediction of BCR by this inclusion of the RNA signature was confirmed, as the corresponding curves generally ran above the individual curves of the clinicopathological-based tools (Figure S4).

## 3. Discussion

In this retrospective study with three working phases (Figure 1), we identified differentially expressed circRNAs in PCa tissue samples using microarray analysis, performed an analytical validation of eight selected circRNAs and their linear counterparts via RT-qPCR measurements, and successfully elaborated RNA-signatures as discriminative biomarkers to differentiate between normal and cancerous PCa tissue and as predictive BCR biomarkers. This information was combined with clinicopathological variables to improve the prediction of BCR.

For the genome-wide identification of circRNAs in PCa tissue, we used six paired PCa tissue samples in the discovery phase in a microarray approach. Generally, microarray analysis is considered a strong and reliable tool for predicting circRNA profiles in clinically relevant tissue samples [83]. However, compared with high-throughput circRNA sequencing analysis with its discovery potential for new cirRNAs, microarray platforms have the drawback of only including a limited number of already validated circRNAs [84]. The microarray analysis identified 43 upregulated and 134 downregulated circRNAs with a higher than absolute 1.5-fold change in malignant tissue samples (Figure 2C). For further evaluation, we chose three upregulated (circ*LPP*, circ*STIL*, and circ*NEAT1*) and three downregulated (circ*ATXN10*, circ*CRIM1*, and circ*RHOBTB3*) circRNAs based on their differential expression levels identified in the microarray analysis and in an in silico circRNA-miRNA-gene interaction analysis (Supplementary Information S2 (Supplementary Materials) with Figure S1). In addition, circ*CSNK1G3* and circ*GUCY1A2* were included in this panel for control purposes, since they had already been investigated by other working groups at the start of our study [44,45]. Furthermore, in order to correlate our results with the linear products of the circRNA host genes in an integrative approach, we included the linRNA counterparts of the circRNAs in this study.

After successful experimental validation of the circular feature of the selected circRNAs and the establishment of their fit-for-purpose RT-qPCR assays (Section 2.3), a comparison of the microarray and RT-qPCR data of the eight circRNAs showed discrepant results for circ*STIL* and circ*LPP* (Table 4). For the other examined circRNAs, the results of the RT-qPCR and microarray analyses were found to be congruent. The downregulation of circ*STIL* and circ*LPP* in the malignant vs. adjacent normal PCa tissue samples was confirmed via RT-qPCR measurements of the 79 paired samples (Figure 4). Inconsistent differential expression of circRNAs in microarray or sequencing vs. in the RT-qPCR analyses has also been observed in previous studies of PCa and other cancers. Shan et al. [85] identified consistent expression between microarray and RT-qPCR analyses in four of five selected circRNAs using 90 PCa and paired non-cancerous tissue samples. Yan et al. [86] reported that three of four selected circRNAs in PCa cells analyzed by RT-qPCR showed consistent high-throughput sequencing results. Qui et al. [87] found an upregulation of circ*CASP8AP2* in hepatocellular carcinoma compared to adjacent normal tissue by sequencing, but in RT-qPCR analyses, this circRNA was downregulated. The reason for these discrepancies between microarray/sequencing data and RT-qPCR results is not clear. Since the same samples were used for the different analytical techniques, it can be assumed that there can only be analytical or post-analytical reasons for these results [31]. The use of different normalization approaches, dependence on the digestion effect of RNase R on circRNAs and linear mRNAs, and method-dependent effect of RNA integrity on the measurement results might be the reasons for these discrepancies [88,89,90,91]. Considering these aspects, the expression evaluation of circRNAs in isolated total RNA samples using RT-qPCR measurements combined with validated cancer-specific reference genes, as done in the present study, might be a practical way to minimize such discrepancies [88]. Further research is needed in this respect, but this was beyond the scope of this study. Furthermore, regarding these discrepancies, the inconsistent results reported for the same circRNAs when examined in different studies should be mentioned. For example, Kong et al. [92] found that *hsa*_circ_0006404 was upregulated in 53 paired PCa samples. In contrast, Shen et al. [48] showed that the same circRNA was downregulated in 22 low-grade and 22 high-grade PCa tissue samples in comparison with 18 normal prostate tissue samples. In addition to the above-mentioned possible analytical and post-analytical reasons, different clinicopathological characteristics of the investigated study cohorts, but also pre-analytical interferences due to the different “quality” of the tissue samples used in different studies may be responsible for these differences [88].

The comparison of the expression levels of circRNAs and linRNAs in the 79 paired PCa tissue samples revealed interesting relationships (Figure 4). Here, we found that all linRNAs except for lin*CSNK1G3* had significantly higher expression levels than the circular RNAs. This observation was concordant with results shown in earlier circRNA studies [23,24,93]. Additionally, circ*STIL* showed significantly lower expression in tumor samples than in normal tissue samples, while linSTIL was significantly higher expressed in tumor samples. Moreover, the normalized expression of lin*NEAT1* was nearly 17,000-fold higher than the expression level of circ*NEAT1* (Figure 4 and Table S10), while the ratio between tumor and normal tissue was equal for both RNAs (circ*NEAT1*: +1.70 vs. lin*NEAT1*: +1.75). A possible explanation for the high linRNA expression could be the similarly abundant expression of the lncRNA *NEAT1* (lnc*NEAT1*) in PCa, which may be an indicator of the independent expression of circRNAs and linRNAs/lncRNAs [94,95]. However, because of the limited performance data from the circ*NEAT1* RT-qPCR analyses and the incomplete detection of this analyte in all samples, we did not include this circRNAs in the multivariable analyses. Nevertheless, more analytically sensitive quantification techniques like the droplet digital polymerase chain reaction should be used to allow this circRNA to be included in future studies.

The differential expression of the malignant and adjacent normal tissue samples identified circ*ATXN10* and lin*STIL* as strong biomarkers in terms of differentiating between tumor and normal tissue. The AUC value for these two markers combined was 0.892 (95% CI: 0.834–0.925). The decision curve analysis also showed a higher discriminative ability when combining circ*ATXN10* and lin*STIL*, compared with applying them alone (Figure 5). Xia et al. [47] evaluated the diagnostic potential of the two circRNAs circ_0057558 and circ_0062019 in PCa. When applying these two circRNAs in combination, an AUC value of 0.861 was achieved. Another working group identified *hsa*_circ_0001633, *hsa*_circ_0001206, and *hsa*_circ_0009061 as possible discriminative tissue biomarkers with respective AUCs of 0.809, 0.774, and 0.711 [58]. Although these results are promising, the ability of circRNAs to differentiate between malignant and non-malignant tissue in other cancer types is much higher. In a recent study on circRNAs in clear cell renal cell carcinoma, we identified circ*EGLN3* (*hsa*_circ_0101692) as a strong marker for differentiation between normal and cancerous tissue, with an AUC of 0.98 when used alone and an AUC of 0.99 when combined with its linear counterpart [22]. Nevertheless, in the future, circRNAs might be used as components of a molecular pattern to improve diagnostic accuracy in the pathological evaluation of cancerous tissue and to provide possible helpful information on the development processes of cancer.

The associations of circRNAs and linRNAs with each other and with standard clinicopathological variables is of special interest. The following distinctive features were noteworthy: (i) the expressions of all circRNAs were found to be strongly correlated in both the adjacent normal and malignant tissue samples, except for circ*NEAT1* and partly for circ*RHOBTB3*, while this correlation feature was mainly lost between paired samples (Tables S12–S14); (ii) different correlation coefficients between circRNAs and the linear transcripts were observed in paired malignant and normal tissue samples (Table S15); and (iii) all circRNAs except circ*NEAT1* and circ*RHOBTB3* were significantly correlated with the ISUP grade but not with other relevant clinicopathological variables such as preoperative PSA, the tumor stage, and the surgical margin (Table S11). Thus, in comparison with other studies that examined other circRNAs [44,51,58,59,60,61,62], few associations with generally relevant clinicopathological PCa variables were identified. This is by no means a primary disadvantage with regard to their potential clinical validity as prognostic/predictive markers. In contrast, this expression of RNAs mostly independent from clinicopathological variables and the other mentioned particular correlations is a key characteristic of orthogonal biomarkers [96]. Biomarkers of this kind are a real prerequisite for gaining information additional to that derived from established variables and for improving, for example, the prediction accuracy of a clinical outcome endpoint [97].

In the introduction, we described the aim of this study as being to evaluate the clinical validity of circRNAs and their linear transcripts with regard to BCR after radical prostatectomy. The essential problems in this respect were explained and need not be repeated, but it should be stressed that data on circRNAs and BCR are lacking. It was therefore particularly important to follow the REMARK guidelines, which recommend the use of continuous expression data in Cox regression analyses as predictive variables of an endpoint and the rejection of primary dichotomized data applications [82]. This study investigated the ability of the circRNAs and linear transcripts to predict BCR occurrence alone and in combination with clinicopathological variables in a step-by-step process. Circ*ATXN10,* circ*GUCY1A*, and circ*LPP* remained after a multivariable Cox regression analysis of the examined circRNAs with backward elimination in a model of BCR prediction (Table 6). On the other hand, in combination with the linear RNAs, these circRNAs were eliminated in the multivariable Cox regression analysis and thus were found to have no role in BCR prediction in comparison to the linRNAs. Only lin*GUCY1A2*, lin*NEAT1*, and lin*STIL* were identified as relevant BCR predictor variables, and these were subsequently termed the “RNA signature” (Table 7). This result is by no means surprising. By comparing circRNA expression with the expression of linear counterparts, the independent clinical value of circRNAs and linear transcripts has already been reported in other studies [44,98]. Therefore, it makes sense to take this functional aspect into account in an integrative approach by simultaneously determining circRNAs and their linear transcripts. Loss of information could thus be avoided. However, this requires that RT-qPCR determinations, as in our study, are performed on isolated total RNA without RNase R pretreatment and that validated reference genes are used to normalize relative expression quantities. This problem was also recently highlighted when the database MiOncoCirc was introduced [39]. The authors recommended the use of a special capture exome RNA-sequencing protocol without RNase R pretreatment [99] in order to determine the actual relationship between circRNA and linRNA in the tissue [39].

Furthermore, the linRNAs were evaluated as BCR predictors in a univariable Cox regression analysis with the TCGA dataset (Table S17). Especially noteworthy was lin*STIL*, which was found to be significantly associated with the risk of BCR. In contrast, circ*CSNK1G3*, which was selected by Chen et al. [44] as an example for demonstration of its functional mechanisms, was not found to be a relevant BCR predictor in our study (Table 6) or in the TCGA dataset (Table S17).

To assess the clinical validity of this final RNA signature, we compared the C-statistic data of the prognostic indices of the RNA signature with those of four established models frequently used in clinical practice and our developed model based on using only clinicopathological variables. The C-statistic data of the RNA signature did not differ from those of established clinical models, except for the model developed by D’Amico et al. [10], which showed statistically significantly lower values. However, most importantly, when the clinical models were combined with the RNA signature, statistically significant improvements in the BCR predictive accuracy or at least corresponding tendencies were evident (Table 9). This improved predictive accuracy was confirmed by decision curve analyses (Figure S4). Decision curve analysis has been postulated as the most informative metric for an incremental predictive benefit [100]. These results support the view that there is considerable potential for improvement of the current prognostic models based only on clinicopathological factors by including molecular RNA markers [17,18,19,20,21]. Recently, the NCCN Prostate Cancer Guideline Panel suggested that tissue-based tests like Decipher, OncoType, Dx Prostate, Prolaris, and ProMark could be considered for initial PCa risk assessment [101].

Of the 16 RNAs examined, a total of six RNAs were represented in the combined models after ROC and multivariable Cox regression analyses (Table 5 and Table 6). These were circ*ATXN10* and lin*STIL* as tissue differentiation markers and BCR predictors (Table 5 and Table 6), and circ*GUCY1A2* and its linear counterpart, circ*LPP*, and lin*NEAT1* for BCR prediction. As explained above, ultimately only lin*GUCY1A2*, lin*NEAT1*, and lin*STIL* remained in the final model as the RNA signature for BCR prediction. So far, there are few data on the listed RNAs in the context of PCa and other cancers. Expression data that could be used for the differentiation of tissue samples as well as BCR markers are missing. Reference to possible biological functions has only been made in few cases, and the validity as a biomarker has only been considered for lin*NEAT1* [95]. Since our intention in this study was primarily to investigate the clinical validity of the selected RNAs as possible biomarkers, we deliberately refrained from undertaking functional investigations. Furthermore, the clinical validity of a marker and thus its applicability in a clinical setting is not primarily linked to its functional significance [102]. The development of an applicable biomarker should focus on demonstrating a benefit in comparison to the methods used to date [79]. To formulate this opinion in exaggerated terms, only proof of the meaningful use of a biomarker for a specific clinical problem should be a justified reason to characterize its possible biological background experimentally. Thus, a brief summary of the current state regarding the biological backgrounds of the relevant RNAs identified herein as potential biomarkers is given to provide directions for future work.

In detail, this is as follows:

Circ*ATXN10* has not yet been discussed in connection with cancer. Our data here represent the first results in this area.

Circ*GUCY1A2* was found to be of particular importance in PCa pathogenesis in an investigation of various PCa cells based on differential expression and bioinformatics information [45]. Experimental findings and data on human PCa tissue samples, as well as on lin*GUCY1A2*, are not yet available. In this respect, our data represent the first information in this area.

For lin*STIL*, Wu et al. [63] found increased expression in PCa tissue, similar to the results of our study. In cell experiments, these lin*STIL* changes were shown to be responsible for stimulating the proliferation of PCa cells and suppressing apoptosis through interactions with various signaling pathways. An investigation on gastric cancer confirmed these effects of upregulated lin*STIL* [103].

Circ*LPP* has not yet been investigated in PCa or other cancers regarding to its biological functions or its potential as a biomarker. Reduced expression of the host gene has been described in lung cancer, similar to the results of the present PCa study [66]. Cell culture experiments on myeloma cells showed that a loss of *LPP* leads to the upregulation of N-cadherin, subsequently promoting tumor cell invasion and metastasis through epithelial-mesenchymal transition.

Lin*NEAT1* has been described in some studies as upregulated mRNA with different oncogenic effects on PCa cells. In PCa, it promotes the expression of the oncogene *HMGA2* through the sponging of miR-98-5p, as well as leading to docetaxel resistance by sponging miR-34a-5p and miR-204-5p [65,104]. Furthermore, *NEAT1* promotes the proliferation of PCa cells in connection with the steroid receptor co-activator (*SCRC3*) through the insulin-like growth factor 1 receptor/AKT serine/threonine kinase 1 (*IGF1R/AKT*) signaling pathway [105]. As shown in the present study using BCR as the clinical endpoint, Bai et al. [95] reported increased expression of *NEAT1* mRNA as being an independent prognostic factor for overall patient survival.

Despite our efforts to make this study as comprehensive and bias-free as possible, particularly taking into account the REMARK, MIQE, and STARD guidelines, it had inherent limitations. These include the retrospective nature of this study, the lack of external validation, and the choice of BCR as an endpoint without consideration of alternative clinical endpoints such as metastasis-free survival or cancer-specific survival. On the other hand it should be emphasized that our data calculated with the bootstrapping method as preferable approach for internal validation [106] confirmed the reliability of results obtained with the constructed models in this study.

## 4. Materials and Methods

### 4.1. Patients and Tissue Samples

The Ethics Committee of the Charité-University Medicine Berlin approved the study (EA1/134/12; approval date: 22 June 2012). Informed consent was obtained from all patients. The study was performed in accordance with the Declaration of Helsinki. Corresponding study guidelines (Minimum Information for Publication of Quantitative Real-Time PCR Experiments (MIQE), Updated List of Essential Items for Reporting Diagnostic Accuracy Studies (STARD 2015), and Reporting Recommendations for Tumor Marker Prognostic Studies (REMARK)) were taken into account [76,79,82].

Tissue samples from PCa patients undergoing radical prostatectomy were snap-frozen in liquid nitrogen immediately after surgery and stored at −80 °C or were immediately transferred into RNAlater stabilization reagent (Qiagen, Hilden, Germany) and stored at −20 °C until RNA isolation as described previously [81,107,108]. Tumor staging and grading (Table 1) was reviewed by two experienced uropathologists (E.K., S.E.) according to the criteria of the International Union against Cancer (UICC TNM, 8th edition) and the World Health Organization/International Society of Urological Pathology (WHO/ISUP) [109,110], respectively.

### 4.2. Analytical Methods

#### 4.2.1. Total RNA Samples and Their Characteristics

Total RNA was isolated from tissue pieces of 31 mg (median value, 95% CI: 30–32) collected from the abovementioned preserved tissue specimens using a special punch-bioptic technique as reported in our previous publications [108,111,112]. This procedure allows to obtain tumor tissue (>90%) and matched normal tissue completely free of tumor filtrates and without inflammation or atrophy. Prominent inflammatory infiltrates, lack of epithelium due to stromal hyperplasia, and prostatic intraepithelial neoplasia were used as exclusion criteria. Taking into account these criteria, a largely bias-free comparison of the expression data between the adjacent normal and malignant tissue samples can be considered. The miRNeasy Mini Kit (Qiagen, Hilden, Germany) with an on-column DNA digestion step, according to the producer’s instructions, was used for total RNA isolation [107,108,112]. Spectrophotometric quantification and quality assessment of the total RNA samples were performed using the NanoDrop 1000 Spectrophotometer (NanoDrop Technologies, Wilmington, DE, USA) and the Bioanalyzer 2100 with the Agilent RNA 6000 Nano Chip Kit (Agilent Technologies, Santa Clara, CA, USA), as detailed reported in our previous publications [81,107,108]. The RNA samples, isolated with 30 µL nuclease-free water, showed the following characteristics: a median absorbance ratio at 260 to 280 nm of 2.12 (95% CI: 2.12 to 2.13), a median absorbance ratio at 260 to 230 nm of 1.99 (95% CI: 1.97 to 2.03), a median RNA integrity number (RIN) value of 7.00 (95% CI: 6.90 to 7.20), and a median RNA concentration of 1096 ng/µL (95% CI: 1001 to 1214). RNA samples were stored at −80 °C. Further details are listed in the checklist of the MIQE guidelines (Table S1).

#### 4.2.2. Microarray Detection of circRNAs

Using isolated total RNA samples from six paired adjacent normal and malignant tissue samples of PCa specimens (1× pT3a with ISUP 2, 1× pT3b with ISUP 2, 2× pT3a with ISUP 3, 2× pT3b with ISUP 3), microarray analyses were performed as a custom order by ArrayStar Inc. (Rockville, MD, USA), as previously reported [22]. Briefly, RNA samples were digested with RNase R to destroy linear RNAs and enrich circular RNAs. Afterwards, the circRNAs were amplified, transcribed, fluorescently labeled, and hybridized on the ArrayStar Human Circular RNA Array. This array is designed to detect 13,617 circRNAs. The Agilent scanner (G2505C) and softwares (Agilent Feature Extraction software version 11.0.1.1 and Agilent GeneSpring GX) were used for imaging scanning and analysis. Quantile normalization was used to normalize the obtained probe intensities. The R Bioconductor “limma” package was applied to calculate the differential expression between the matched pairs. All data were compiled in the accompanying separate Excel file with all additional information and annotation details (Supplementary Microarray Data File.xlsx (Supplementary Materials)).

#### 4.2.3. RT-qPCR Methodology and circRNA Validation Methods

RT-qPCR measurements were performed according to the recommendations in the MIQE guidelines [76]. The corresponding comments are listed in the abovementioned checklist of the MIQE guidelines and applied for all assays (Supplementary Information S3 with Table S1 and the additional Tables S2–S7).

Detailed validation procedures based on the general characteristics of circRNAs regarding their resistance to RNase R digestion, their lack of a poly(A) tail using separate reverse transcription with random hexamer and oligo(dT)_18_ primers, and the proof of the backsplice junctions by Sanger sequencing, were described in our previous report on circRNAs in kidney cancer [22] and are briefly summarized in Supplementary Information S4 to explain the data in Figure 3. A melting curve analysis and gel electrophoresis were additionally carried out as confirmatory approaches to verify the analytical specificity of the RT-qPCR products of all circRNAs (Figures S2 and S3).

The Maxima First Strand cDNA Synthesis Kit for RT-qPCR (Thermo Fisher Scientific, Waltham, MA, USA; Cat.No. K1642) was used for cDNA synthesis of circRNAs and their linear counterparts, as this kit contains a ready-to use mixture of random hexamer and oligo(dT)_18_ primers (Table S2A). For the validation of circRNAs, we addressed the issue of reliability of reverse transcription using another cDNA synthesis kit (Transcriptor First Strand cDNA Synthesis Kit, Life Science Roche, Mannheim, Germany; Cat. No. 04379012001) that allows separate priming with either random hexamer or oligo(dT)_18_ primers (Table S2B).

The LightCycler 480 Instrument (Roche Molecular Diagnostics, Mannheim, Germany) with white 96-well plates (Cat.No. 04729692001) and a reaction volume of 10 µL was used for all real-time qPCR runs. The Maxima SYBR Green qPCR Master Mix (Thermo Fisher Scientific; Cat.No. K0252) was used. Primers were designed using the blasting tool provided by Primer3 [113] and synthesized by TIB MOLBIOL GmbH (Berlin, Germany). The reaction conditions with the list of primers, measurement details, setup of the assays, and performance data for all eight circRNAs and their linear counterparts as well as the reference genes *ALAS1* and *HPRT1* as a combined pair for normalizing expression data in PCa samples [81] are compiled in Table S6 with the protocols A–I. No-template controls and no-reverse transcriptase controls were always included and showed negative results. All cDNA samples were measured at least in duplicate and the resulting mean values of the quantification cycles were used for further calculations.

The software qBase+ version 3.2 (Biogazelle, Zwijnaarde, Belgium; www.qbaseplus.com) was used for Cq data evaluation [114,115]. This program is based on a generalized model of the 2^−ΔΔCq^ approach with correction of the amplification efficiency. Cq values were converted into relative quantities (RQs) with respect to equal amounts of total RNA for all samples used for the cDNA synthesis, and they were converted into normalized relative quantities (NRQs) based on the expression of the two cancer-specific reference genes mentioned above, *ALAS1* and *HPRT1*.

### 4.3. Statistics and Data Analysis

The statistical programs SPSS Version 25 (IBM Corp., Armonk, NY, USA) with the bootstrap module, GraphPad Prism version 8.4.3 (GraphPad Software, La Jolla, CA, USA), and MedCalc version 19.4. (MedCalc Software bvba, 8400 Ostend, Belgium) with bootstrapping C-statistics were used. *p* < 0.05 (two-sided) represented statistical significance. The Mann-Whitney *U*-test, Wilcoxon test, *t*-test, and Spearman rank correlation coefficients were used for continuous data and Chi-squared or Fisher’s exact tests were used for categorical data. Univariable and multivariable Cox proportional hazard regression analyses were used for survival analysis of the endpoint BCR. C-statistic values based on ROC analyses with AUC calculation of prognostic indices of Cox regression analyses and corresponding decision curve analyses were determined to characterize the discrimination/prediction capacity of the different variables and models [116,117,118,119]. Sample size and power calculations were performed using the programs GPower version 3.1.9.4 [120], GraphPad StatMate version 2.0 (GraphPad Software), and MedCalc version 19.4 (MedCalc Software bvba), and the results were used to design the study. The prediction tool CircInteractome [69] was used for the in silico analysis of circRNAs to identify potential miRNA-gene interactions using the miRDB and TargetScan databases [70,71]. TCGA-PRAD RNAseq data were downloaded and analyzed with R (version 3.6) using the “TCGA2stat” library and the “survival” library for univariable Cox regression analyses of the linear counterparts of the eight circRNAs.

## 5. Conclusions

This study investigated the value of circRNAs and their linear counterparts as potential diagnostic and prognostic biomarkers in PCa using a genome-wide, integrative, and exploratory approach. We showed that the combination of circ*ATXN10* and lin*STIL* provides a strong marker pair that can be used to discriminate between normal and malignant PCa tissue samples. Furthermore, we identified lin*GUCY1A2*, lin*NEAT1*, and lin*STIL* as potentially useful prognostic biomarkers to increase the accuracy of BCR prediction in PCa patients in combination with standard risk prediction models based only on clinicopathological variables. These results support the thesis that there is considerable potential to improve the current clinical prognostic models by including molecular RNA markers. In future studies, it will be advantageous to include circRNAs into the clinicogenomic models in addition to the established RNA classes such as miRNA, mRNA, piwiRNA or lncRNA.

## Figures and Tables

**Table 1:** Clinicopathological characteristics of the study group.

Characteristics	All Patients	Patients with Biochemical Recurrence	Patients without Biochemical Recurrence	*p*-Value ^a^
Patients, no. (%)	115 (100)	39 (34)	76 (66)	
Age, median years, (IQR)	67 (62–70)	66 (59–71)	67 (64–70)	0.339
PSA, µg/L (IQR)	7.7 (5.4–12.2)	9.7 (6.1–19.5)	7.0 (5.4–9.5)	0.011
Prostate volume, cm^3^ (IQR)	32 (25–45)	30 (23–39)	33 (26–45)	0.264
DRE, no. (%)				0.067
Non-suspicious	67 (58)	17 (44)	50 (66)	
Suspicious	32 (28)	14 (36)	18 (24)	
Unclassified	16 (14)	8 (20)	8 (10)	
pT status, no. (%)				0.005
pT1c	1 (1)	0	1 (1)	
pT2a	2 (2)	0	2 (3)	
pT2b	1 (1)	0	1 (1)	
pT2c	61 (53)	13 (33)	48 (63)	
pT3a	27 (23)	11 (28)	16 (21)	
pT3b	23 (20)	15 (39)	8 (11)	
ISUP Grade groups, no. (%)				0.0001
1	26 (23)	2 (5)	24 (31)	
2	47 (41)	13 (33)	34 (45)	
3	30 (26)	14 (36)	16 (21)	
4	4 (3	4 (11)	0 (0)	
5	8 (7)	6 (15)	2 (3)	
pN status, no. (%)				0.017
pN0/Nx	109 (95)	34 (87)	75 (99)	
pN1	6 (5)	5 (13)	1 (1)	
Surgical margin, no. (%)				
Negative	64 (56)	16 (41)	48 (63)	0.030
Positive	51 (44)	23 (59)	28 (37)	
Follow-up after surgery				<0.0001
Median months (IQR)	41 (26–72)	19.9 (9.8–41)	52 (38–80)	

Abbreviations: CI, confidence interval; DRE, digital rectal examination; IQR, interquartile range; ISUP Grade groups, histopathological grade system based on Gleason score according to the International Society of Urologic Pathology; pN, lymph node status; PSA, total prostate specific antigen before surgery; pT, pathological tumor classification. ^a^ *p*-Values (Mann-Whitney *U* test; Chi-square or Fisher’s exact test) indicate the association of the clinicopathological variables with patients with and without biochemical recurrence.

**Table 2:** List of circular RNAs (circRNAs) seleceted for further evaluation in this study based on their differential expression between malignant and adjacent normal tissue samples data in the microarray discovery study phase and literature search.

circRNA in Manuscript	circRNA ID in circBase ^a,b^	Absolute Fold Change on Microarray (*p*-Value)	Best Transcript	Official Gene Symbol (Official Gene Name)
Upregulated circRNAs				
circ*LPP*	circ_0003759	1.94 (0.025)	NM_005578.5	*LPP* (LIM domain containing preferred translocation partner in lipoma)
circ*NEAT1*	circ_0000324	2.73 (0.0235)	NR_131012.1	*NEAT1* (Nuclear paraspeckle assembly transcript 1)
circ*STIL*	circ_0000069	1.75 (0.007)	NM_001282936.1	*STIL* (STIL centriolar assembly protein)
Downregulated circRNAs				
circ*ATXN10*	circ_0001246	2.48 (0.001))	NM_013236.4	*ATXN10* (Ataxin 10)
circ*CRIM1*	circ_0007386	2.17 (0.006))	NM_016441.3	*CRIM1* (Cysteine rich transmembrane BMP regulator 1)
circ*RHOBTB3*	circ_0007444	2.14 (0.0003)	NM_014899.4	*RHOBTB3* (Rho related BTB domain containing 3)
circRNAs from Literature ^c^				
circ*CSNK1G3*	circ_0001522	−1.31 (0.003)	NM_001044723.2	*CSNK1G3* (Casein kinase 1 gamma 3)
circ*GUCY1A2*	circ_0008602	−1.02 (0.305)	NM_000855.3	*GUCY1A2* (Guanylate cyclase 1 soluble subunit alpha 2)

^a^ The obligatory prefix *hsa*_ was omitted to facilitate the readability. ^b^ In the separate Supplementary Microarray Data File.xlsx as part of the Supplementary Materials, detailed information is given for all detected circRNAs including source, chromosome localization, strand, circRNA type, sequences, and the circRNA IDs specific for ArrayStar Microarrays and the database circBase [75]. ^c^ Chen et al. [44] for circ*CSNK1G3* and Zhang et al. [45] for circ*GUCY1A2*.

**Table 3:** Repeatability and reproducibility of RT-qPCR measurements.

RNA	Repeatability ^a^		Reproducibility ^b^	
	Cq Value Mean (%RSD)	Relative Quantities Mean (%RSD)	Cq Value Mean ± SD (%RSD)	Relative Quantities Mean ± SD (%RSD)
circ*ATXN10*	24.49 (0.595)	1.345 (10.4)	24.31 ± 0.144 (0.591)	1.004 ± 0.100 (9.98)
circ*CRIM1*	24.61 (0.455)	1.299 (7.59)	24.39 ± 0.115 (0.472)	1.003 ± 0.078 (7.79)
circ*CSNK1G3*	21.47 (0.289)	1.164 (4.28)	21.34 ± 0.131 (0.613)	1.003 ± 0.093 (9.22)
circ*GUCY1A2*	24.68 (0.516)	1.461 (8.81)	24.68 ± 0.134 (0.541)	1.003 ± 0.092 (9.18)
circ*LPP*	25.71 (0.314)	1.177 (5.71)	25.76 ± 0.104 (0.406)	1.002 ± 0.070 (7.00)
circ*NEAT1*	35.56 (0.680)	1.285 (16.4)	36.80 ± 0.309 (0.838)	1.017 ± 0.214 (21.1)
circ*RHOBTB3*	23.91 (0.241)	1.055 (3.95)	24.02 ± 0.178 (0.739)	1.006 ± 0.121 (12.1)
circ*STIL*	28.51 (0.542)	1.261 (10.9)	28.47 ± 0.105 (0.369)	1.002 ± 0.0.72 (7.18)
lin*ATXN10*	20.23 (0.341)	1.250 (5.07)	20.21 ± 0.106 (0.525)	1.002 ± 0.072 (7.14)
lin*CRIM1*	21.67 (0.257)	1.305 (3.85)	21.49 ± 0.145(0.673)	1.004 ± 0.102 (10.1)
lin*CSNK1G3*	21.73 (0.275)	1.052 (4.13)	22.23 ± 0.152 (0.683)	1.003 ± 0.091 (9.08)
lin*GUCY1A2*	23.55 (0.480)	1.458 (8.22)	22.51 ± 0.134 (0.596)	1.004 ± 0.096 (9.57)
lin*LPP*	19.27 (0.472)	1.193 (6.64)	19.06 ± 0.121 (0.633)	1.003 ± 0.085 (8.46)
lin*NEAT1*	18.79 (0.231)	1.641 (2.96)	19.80 ± 0.079 (0.401)	1.001 ± 0.054 (5.38)
lin*RHOBTB3*	21.23 (0.259)	1.147 (3.73)	21.34 ± 0.170 (0.796)	1.006 ± 0.120 (11.9)
lin*STIL*	25.88 (0.411)	1.381 (5.22)	26.22 ± 0.131 (0.500)	1.003 ± 0.089 (8.94)
*ALAS1*	23.04 (0.305)	1.113 (4.86)	23.32 ± 0.064 (0.275)	1.001 ± 0.043 (4.33)
*HPRT1*	25.32 (0.411)	1.192 (7.09)	25.97 ± 0.112 (0.432)	1.002 ± 0.077 (7.75)

Abbreviations: Cq, quantification cycle; %RSD, percent relative standard deviation; SD, standard deviation; *ALAS1*, 5′-aminolevulinate synthase 1; *HPRT1*, hypoxanthine phosphoribosyltransferase 1. *ALAS1* and *HPRT1* were used as reference genes [81]. ^a^ *n* = 20; %RSD was calculated from duplicate measurements using the root mean square method based on Cq values and relative quantities, respectively. ^b^ *n* = 5 inter-assay measurements; %RSD (Cq) corresponds to the percent relative standard deviation using the Cq values. %RSD (Relative quantities) corresponds to the percent relative standard deviation using the relative quantities within the inter-assay measurements of the respective RNA variable.

**Table 4:** Comparison of the circRNA expression data of the six paired tumor and adjacent normal tissue samples used in the microarray and RT-qPCR analyses.

circRNA	Microarray Expression Data ^a^	RT-qPCR Expression Data ^b^
	Ratio of Tumor to Normal Tissue (*p*-Value)	Ratio of Tumor to Normal Tissue (*p*-Value)
circ*ATXN10*	−2.48 (0.001)	−2.09 (0.020)
circ*CRIM1*	−2.17 (0.006)	−2.45 (0.027)
circ*CSNK1G3*	−1.31 (0.003)	−1.84 (0.027)
circ*GUCY1A2*	−1.02 (0.305)	−1.07 (0.781)
**circ*LPP***	**+1.94 (0.025)**	**−2.01 (0.004)**
circ*NEAT1*	+2.73 (0.024)	+4.33 (0.061)
circ*RHOBTB3*	−2.14 (0.0003)	−2.05 (0.041)
**circ*STIL***	**+1.75 (0.007)**	**−1.35 (0.086)**

^a^ Expression data correspond to the data shown in Table 2 (*t*-test of the six paired tissue samples used in microarray analyses). ^b^ Expression data of the paired samples used in the microarray analyses measured by the established circRNA assays in this study and normalized to the reference genes *ALAS1* and *HPRT1* (*t*-test of paired data).

**Table 5:** Receiver operating characteristic (ROC) curve analyses of circRNAs and their linear transcripts for discrimination between adjacent normal (*n* = 79) and malignant (*n* = 115) tissue samples from prostate cancer specimens. For circ*NEAT1*, only 118 samples could be analyzed.

RNAs	AUC (95% CI)	*p*-Value Different to AUC = 0.5	Differentiating Ability at the Youden Index ^a^		Overall Correct Classification (%)
			Sensitivity (95% CI)	Specificity (95% CI)	
**Single variable**					
circ*ATXN10*	0.801 (0.719–0.851)	<0.0001	77 (68–84)	72 (61–82)	74.2
lin*ATXN10* (*p* < 0.0001) ^b^	0.525 (0.442–0.606)	0.534	45 (36–55)	65 (53–75)	59.3
circ*CRIM1*	0.743 (0.660–0.808)	<0.0001	74 (66–82)	66 (54–76)	67.0
lin*CRIM1* (*p* = 0.143) ^b^	0.778 (0.697–0.836)	<0.0001	76 (67–83)	76 (65–85)	71.7
circ*CSNK1G3*	0.780 (0.715–0.836)	<0.0001	69 (59–77)	77 (66–86)	72.7
lin*CSNK1G3* (*p* < 0.0001) ^b^	0.518 (0.436–0.602)	0.661	49 (40–59)	59 (48–70)	59.3
circ*GUCY1A2*	0.545 (0.459–0.624)	0.285	65 (56–74)	48 (37–60)	58.8
lin*GUCY1A2* (*p* = 0.208) ^b^	0.583 (0.493–0.665)	0.051	70 (60–78)	49 (40–61)	58.3
circ*LPP*	0.773 (0.708–0.830)	<0.0001	71 (62–79)	75 (64–84)	72.2
lin*LPP* (*p* = 0.321) ^b^	0.762 (0.696–0.820)	<0.0001	70 (61–79)	76 (65–85)	71.6
circ*NEAT1*	0.634 (0.552–0.733)	<0.013	72 (60–82)	51 (36–67)	62.5
lin*NEAT1* (*p* = 0.371) ^b^	0.690 (0.608–0.760)	<0.0001	63 (53–72)	72 (61–82)	63.4
circ*RHOBTB3*	0.684 (0.613–0.749)	<0.0001	73 (64–81)	61 (49–72)	66.0
lin*RHOBTB3* (*p* = 0.013) ^b^	0.520 (0.438–0.605)	0.629	45 (36–55)	67 (56–77)	59.3
circ*STIL*	0.645 (0.556–0.719)	<0.003	53 (44–62)	72 (61–82)	62.9
lin*STIL* (*p* < 0.0001) ^b^	0.841 (0.804–0.912)	<0.0001	78 (70–85)	86 (77–93)	80.4
**Optimized combination**					
circ*ATXN10* + lin*STIL* ^c^	0.892 (0.834–0.925)	<0.0001	79 (71–86)	87 (78–94)	81.4

Abbreviations: AUC, area under the receiver operating characteristic curve; CI, confidence interval. ^a^ The Youden index as a measure of overall diagnostic effectiveness is calculated by (sensitivity + specificity) − 1. ^b^ Significances between the AUC values of individual circRNAs and their linear counterparts. ^c^ Calculated by binary logistic regression using all RNAs in a backward elimination approach. Results are based on bias-corrected and accelerated bootstrap calculation with 2000 iterations.

**Table 6:** Construction of separate tools for prediction of biochemical recurrence using circRNAs and their linear counterparts.

	Univariable Cox Regression ^a^		Multivariable Cox Regression			
RNA			Full Model ^b^		Reduced Model after Backward Elimination ^c^	
	HR (95% CI)	*p*-Value	HR (95% CI)	*p*-Value	HR (95% CI)	*p*-Value
**Circular RNAs**						
circ*ATXN10*	0.39 (0.10–1.88)	0.239	0.27 (0.08–0.89)	0.032	0.31 (0.13–0.76)	0.011
circ*CRIM1*	0.69 (0.22–2.16)	0.521	-	-	-	-
circ*CSNK1G3*	2.32 (0.51–10.6)	0.240	1.96 (0.50–7.68)	0.336	-	-
circ*GUCY1A2*	1.31 (0.98–1.75)	0.065	1.32 (0.99–1.75)	0.051	1.33 (1.02–1.74)	0.037
circ*LPP*	1.86 (0.84–4.12)	0.125	1.76 (0.78–3.96)	0.169	1.89 (0.91–3.95)	0.092
circ*RHOBTB3*	0.86 (0.38–1.93)	0.705	-	-	-	-
circ*STIL*	0.53 (0.18–1.53)	0.238	0.57 (0.21–1.62)	0.293	-	-
**Linear mRNAs**						
lin*ATXN10*	1.23 (0.15–10.2)	0.846	-	-	--	-
lin*CRIM1*	0.90 (0.22–3.76)	0.887	-	-	-	-
lin*CSNK1G3*	0.47 (0.09–2.60)	0.399	-	-	-	-
lin*GUCY1A2*	1.52 (0.99–2.32)	0.050	1.47 (1.09–2.00)	0.012	1.47 (1.09–2.00)	0.012
lin*LPP*	1.06 (0.23–4.76)	0.941				
lin*NEAT1*	1.41 (1.15–1.72)	0.001	1.39 (1.16–1.66)	0.0003	1.39 (1.16–1.66)	0.0003
lin*RHOBTB3*	0.78 (0.20–3.11)	0.727				
lin*STIL*	0.59 (0.32–1.08)	0.086	0.54 (0.30–0.96)	0.037	0.54 (0.30–0.96)	0.037

Abbreviations: HR, hazard ratio; CI, confidence interval. ^a^ As explained in chapter 2.4.1, circ*NEAT1* was excluded from Cox regression analyses. ^b^ The full model included all variables of the univariable Cox regression with hazard ratios of *p*-values < 0.250. ^c^ Reduced model after backward elimination with entry *p* < 0.05 and removal *p* > 0.100. All data of the univariable and final multivariable Cox regression models are calculated by the bias-corrected and accelerated bootstrap method with 2000 resamples.

**Table 7:** Construction of a predictive RNA signature for biochemical recurrence based on Cox regression analysis, using a combination of the separate prediction tools for circRNAs and their linear counterparts.

	Multivariable Cox Regression of the Combined Separate RNA Classifiers			
RNA Prediction Tool	Full Model with all Separate Classifiers ^a^		Reduced Model after Backward Elimination ^b^	
	HR (95% CI)	*p*-Value	HR (95% CI)	*p*-Value
circRNA prediction tool				
circ*ATXN10*	0.45 (0.18–1.12)	0.086	not included	-
circ*GUCY1A2*	0.95 (0.55–1.64)	0.850	not included	-
circ*LPP*	1.37 (0.66–2.82)	0.399	not included	-
linear RNA prediction tool				
lin*GUCY1A2*	1.77 (0.80–3.89)	0.153	1.47 (1.09–2.00)	0.012
lin*NEAT1*	1.33 (1.11–1.60)	0.002	1.39 (1.16–1.66)	0.0003
lin*STIL*	0.52 (0.29–0.94)	0.030	0.54 (0.30–0.96)	0.037

Abbreviations: HR, hazard ratio; CI, confidence interval. ^a^ This model included all six RNA variables indicated in Table 6 as the “Reduced model after backward elimination” of the separate circRNA and linear RNA based prediction tools. ^b^ Reduced model after backward elimination with entry *p* < 0.05 and removal *p* > 0.100. All data of the univariable and final multivariable Cox regression models are calculated by the bias-corrected and accelerated bootstrap method with 2000 resamples.

**Table 8:** Construction of a predictive classifier for biochemical recurrence using Cox regression analyses with clinicopathological variables in 115 patients.

Variable ^a^	Univariable Cox Regression		Multivariable Cox Regression			
			Full Model ^b^		Reduced Model after Backward Elimination ^c^	
	HR (95% CI)	*p*-Value	HR (95% CI)	*p*-Value	HR (95% CI)	*p*-Value
Age	0.97 (0.93–1.02)	0.280				
PSA (> 10 <)	2.24 (1.18–4.18)	0.0130	1.59 (0.83–3.07)	0.162		
DRE	1.24 (0.83–1.95)	0.286				
Margin	2.37(1.24–4.52)	0.009	1.91 (0.98–3.72)	0.056	1.99 (1.03–3.84)	0.041
pN status	2.60 (0.92–7.35)	0.071	0.58 (0.19–1.81)	0.352		
pT stage	2.16 (1.51–3.09)	<0.0001	1.55 (1.03–2.33)	0.037	1.58 (1.05–2.40)	0.030
ISUP Group	1.66 (1.31–2.11	<0.0001	1.55 (1.14–2.10)	0.005	1.43 (1.07–1.91)	0.016

^a^ Abbreviations and stratifications of the variables as indicated in Table 1; CI, confidence interval; HR, hazard ratio. ^b^ The full model included all variables of the univariable Cox regression with hazard ratios of *p* < 0.250. ^c^ Reduced model after backward elimination with entry *p* < 0.05 and removal *p* > 0.100. All data of the univariable and final multivariable Cox regression models are calculated by the bias-corrected and accelerated bootstrap method with 2000 resamples.

**Table 9:** Improved prediction of biochemical recurrence after radical prostatectomy using clinicopathological-based tools in combination with the RNA signature.

Prediction Tool	Clinicopathological-Based Tool	Clinicopathological-Based Tool Combined with RNA Signature	*p*-Value
	AUC (95% CI)	AUC (95% CI)	
*Present study*			
Full model	0.810 (0.726–0.877)	0.841 (0.761–0.902)	0.073
Reduced model	0.804 (0.720–0.872)	0.827 (0.746–0.891)	0.104
*Reference models*			
D’Amico et al. [10]	0.513 (0.418–0.607)	0.718 (0.627–0.798)	0.004
CAPRAS [9]	0.750 (0.660–0.826)	0.799 (0.714–0.868)	0.034
NCCN [11]	0.733 (0.643–0.811)	0.800 (0.715–0.869)	0.035
Stephenson et al. [7]	0.785 (0.699–0.856)	0.821 (0.738–0.886)	0.107

Abbreviations: AUC, area under the receiver operating characteristic curve as C-statistics calculated from the prognostic indices of the Cox regression analyses; CI, confidence interval; CAPRAS, Cancer of the Prostate Risk Assessment Postsurgical Score; NCCN, National Comprehensive Cancer Network; Full model, according to the Cox regression model described in Table 8 with all clinicopathological factors except of age and digital rectal examination; Reduced model, according to the Cox regression model described in Table 8 after backward elimination and finally including only the variables of pT stage, ISUP Group grade, and surgical margin status. Results are based on bias-corrected and accelerated bootstrap calculation with 2000 iterations.

## Supplementary Materials

The following are available online at https://www.mdpi.com/1422-0067/21/21/7812/s1, Supplementary Microarray Data File.xlsx, Supplementary Information S1: Sample size and power calculations, Supplementary Information S2 with Supplementary Figure S1, Supplementary Information S3: RT-qPCR methodology, Supplementary Information S4: CircRNA validation methods, Supplementary Information S5: Associations between circRNAs, linear transcripts and clinicopathological variables, Supplementary Information S6: Cox regressions analyses, C-statistics, and decision curve analyses of BCR prediction tool, Figure S1: Bioinformatic analysis of circRNA–miRNA–gene interaction, Figure S2: Melting curve analysis of circular and linear qPCR products on LightCycler 480, Figure S3: Gel views of analyzed amplicons of (A) cirRNAs and (B) linear RNAs, Figure S4: Decision curve analysis of clinicopathological-based BCR prediction tools, Table S1: MIQE checklist according to Bustin et al., Table S2A: Decision curve analysis of clinicopathological-based BCR prediction tools, Table S2B: cDNA synthesis using Transcriptor First Strand cDNA Synthesis Kit, Table S3: Sequences of amplicons of circRNAs with marked backsplice junctions, primer sequences, and their locations in the host genes, Table S4: QPCR target information of the linear RNAs and the reference genes, Table S5: Primer sequences of circRNAs, linear RNAs, and normalizers, Table S6: Protocols for LightCycler 480 qPCR runs, Table S7: Characteristics of the PCR standard curves, Table S8: Expression ratios of circular and linear RNAs in 79 paired tumor tissue to adjacent normal tissue samples, Table S9: Expression of circRNAs and their linear transcripts in paired adjacent normal tissue samples and malignant tissue samples compared, Table S10: Expression ratios of linear to circular RNAs in paired adjacent normal tissue and prostate carcinoma tissue samples, Table S11: Associations of circRNAs and linear transcripts with clinicopathological variables, Table S12: Spearman rank correlation coefficients between all circRNAs in adjacent normal tissue samples, Table S13: Spearman rank correlation coefficients between all circRNAs in malignant tissue samples, Table S14: Spearman rank correlation coefficients between circRNAs in paired adjacent normal tissue samples and malignant tissue samples, Table S15: Spearman rank correlation coefficients between circRNAs and their linear counterparts in paired adjacent normal tissue and tumor samples, Table S16: C-statistics of prognostic indices of the two RNA-based prediction models, Table S17: Univariate Cox regression data of 8 linear RNAs as BCR predictors of TCGA data, References.

## Abbreviations

%RSD	percent relative standard deviation
*AKT*	AKT serine/threonine kinase 1
*ALAS1*	5′-aminolevulinate synthase 1
*ATXN10*	ataxin 10
AUC	area under the ROC curve
BCR	biochemical recurrence
CAPRAS	Cancer of the Prostate Risk Assessment Postsurgical Score
cDNA	complementary DNA
CI	confidence interval
circ	circular (used in composition with gene symbols to define examined circRNAs)
circRNA	circular RNA
Cq	quantification cycle
*CRIM1*	cysteine rich transmembrane BMP regulator 1
*CSNK1G3*	casein kinase 1 gamma 3
DRE	digital rectal examination
*GUCY1A2*	guanylate cyclase 1 soluble subunit alpha 2
*HPRT1*	hypoxanthine phosphoribosyltransferase 1
HR	hazard ratio
*IGF1R*	insulin like growth factor 1 receptor
IQR	interquartile range
ISUP	International Society of Urologic Pathology
lin	lin (in composition with gene symbols to define examined linRNAs)
linRNA	linear RNA (mRNA)
lnc	long non-coding (used in composition with gene symbols)
*LPP*	LIM domain containing preferred translocation partner in lipoma
MIQE	The Minimum Information for Publication of Quantitative Real-Time PCR Experiments
NCCN	National Comprehensive Cancer Network
*NEAT1*	nuclear paraspeckle assembly transcript 1
NRQ	normalized relative quantity
PCa	prostate cancer
pN	pathological lymph node status
PSA	prostate-specific antigen
pT	pathological tumor classification
REMARK	Reporting Recommendations for Tumor Marker Prognostic Studies
*RHOBTB3*	rho related BTB domain containing 3
RIN	RNA integrity number
ROC	receiver operating characteristic
RQ	relative quantity
RT-qPCR	reverse-transcription quantitative real-time polymerase chain reaction
*SCRC3*	steroid receptor co-activator
STARD	Standards for Reporting of Diagnostic Accuracy Studies
*STIL*	STIL centriolar assembly protein
TCGA (PRAD)	The Cancer Genome Atlas Prostate Cancer
T/N	expression index of circRNA or linRNA in tumor to adjacent normal tissue
TNM	Tumor, Node, Metastases
