# Supplementary material for: Circular RNAs and Their Linear Transcripts as Diagnostic and Prognostic Tissue Biomarkers in Prostate Cancer after Prostatectomy in Combination with Clinicopathological Factors
Source: Int J Mol Sci. 2020 Oct 22;21(21):7812. doi: 10.3390/ijms21217812 (PMC7672590; doi:10.3390/ijms21217812)
Supplement: Supplementary file 1 [file ijms-21-07812-s001.zip › Supplementary Materials_Supplementary Information S1-S6.docx]

**Supplementary Materials**

**Supplementary Microarray Data File.xlsx**

[Supplementary Information S1: Sample size and power calculations - 2 -](#_Toc49798629)

[Supplementary Information S2 with Supplementary Figure S1 - 3 -](#_Toc49798630)

[Figure S1. Bioinformatic analysis of circRNA–miRNA–gene interaction - 3 -](#_Toc49798631)

[Supplementary Information S3: RT-qPCR methodology - 4 -](#_Toc49798632)

[General comments - 4 -](#_Toc49798633)

[Table S1. MIQE checklist according to Bustin et al. [5] - 4 -](#_Toc49798634)

[cDNA synthesis - 9 -](#_Toc49798635)

[Table S2A. cDNA synthesis using the Maxima First Strand cDNA Synthesis Kit - 9 -](#_Toc49798636)

[Table S2B. cDNA synthesis using Transcriptor First Strand cDNA Synthesis Kit. - 10 -](#_Toc49798637)

[qPCR measurements - 10 -](#_Toc49798638)

[Table S3. Sequences of amplicons of circRNAs with marked backsplice junctions, primer sequences, and their locations in the host genes. - 11 -](#_Toc49798639)

[Table S4. QPCR target information of the linear RNAs and the reference genes - 12 -](#_Toc49798640)

[Table S5. Primer sequences of circRNAs, linear RNAs, and normalizers - 13 -](#_Toc49798641)

[Table S6. Protocols for LightCycler 480 qPCR runs - 14 -](#_Toc49798642)

[Performance data of RT-qPCR analyses - 16 -](#_Toc49798643)

[Figure S2: Melting curve analysis of circular and linear qPCR products on LightCycler 480. - 16 -](#_Toc49798644)

[Figure S3. Gel views of analyzed amplicons of (A) cirRNAs and (B) linear RNAs - 17 -](#_Toc49798645)

[Table S7. Characteristics of the PCR standard curves - 18 -](#_Toc49798646)

[Supplementary Information S4: circRNA validation methods - 19 -](#_Toc49798647)

[RNase R digestion of isolated total RNA - 19 -](#_Toc49798648)

[Random hexamer primers vs. oligo(dT)_18_ primers for cDNA synthesis - 19 -](#_Toc49798649)

[Sanger-Sequencing of circRNA backsplice junction - 19 -](#_Toc49798650)

[Supplementary Information 5: Associations between circRNAs, linear transcripts, and clinicopathological variables - 20 -](#_Toc49798651)

[Table S8. Expression ratios of circular and linear RNAs in 79 paired tumor tissue to adjacent normal tissue samples - 20 -](#_Toc49798652)

[Table S9. Expression of circRNAs and their linear transcripts in paired adjacent normal tissue samples and malignant tissue samples compared - 20 -](#_Toc49798653)

[Table S10. Expression ratios of linear to circular RNAs in paired adjacent normal tissue and malignant tissue samples - 21 -](#_Toc49798654)

[Table S11. Associations of circRNAs and linear transcripts with clinicopathological variables - 21 -](#_Toc49798655)

[Table S12. Spearman rank correlation coefficients between all circRNAs in normal tissue samples - 22 -](#_Toc49798656)

[Table S13. Spearman rank correlation coefficients between all circRNAs in malignant tissue samples - 22 -](#_Toc49798657)

[Table S14. Spearman rank correlation coefficients between circRNAs in paired adjacent normal tissue samples and malignant tissue samples - 23 -](#_Toc49798658)

[Table S15. Spearman rank correlation coefficients between circRNAs and their linear counterparts in paired adjacent normal tissue and tumor samples - 23 -](#_Toc49798659)

[Supplementary Information S6: Cox regression analyses, C-statistics data, and decision curve analyses of BCR prediction models - 24 -](#_Toc49798660)

[Table S16. C-statistics of prognostic indices of the two RNA-based prediction models - 24 -](#_Toc49798661)

[Table S17. Univariate Cox regression data of 8 linear RNAs as BCR predictors of TCGA data - 24 -](#_Toc49798662)

[Figure S4. Decision curve analysis of clinicopathological-based BCR prediction tools - 25 -](#_Toc49798663)

**References...........................................................................................................................................................- 26 -**

**Supplementary Information S1**: Sample size and power calculations.

To calculate the necessary sample sizes in this study, we used the statistics programs MedCalc 19.2.0 (MedCalc Software bvba, Ostend, Belgium), GPower 3.1.9.7 (Franz Faul; Kiel University, Kiel, Germany), and GraphPad StatMate 2.0 (GraphPad Software, San Diego, CA, USA). Conventional thresholds were used: α = 5% (significance level) and β = 20% (1-power; power of 80%).

To calculate the necessary sample size according to these specifications in assessing the differential ability of the circRNAs and their linear counterparts between matched pairs of adjacent normal and malignant tissue samples, the receiver operating characteristics (ROC) analysis was used. An area under the ROC curve >0.75 was defined as appropriate discriminative criterion. Under these conditions, at least 19 pairs would be necessary to investigate.

To calculate the necessary sample size to evaluate the validity of variables/markers for the selected clinical endpoint "biochemical recurrence after prostatectomy", a difference of 0.25 in the Kaplan-Meier "survival" rate within a five-year follow up after radical prostatectomy was assumed for useful discrimination. Selecting tissue samples in this retrospective study by a two to one alternate selection process of patients without biochemical recurrence (assuming a rate of 0.90) and with biochemical recurrence (assuming a rate of 0.70), a total sample size of at least 84 would be required.

The final inclusion of 115 patients in this study with total 194 tissue samples (79 pairs of matched adjacent normal and malignant tissue samples and 36 additional malignant tissue samples only) is a sufficiently suitable sample size to perform statistically relevant calculations.

**Supplementary Information S2 with Supplementary Figure S1**


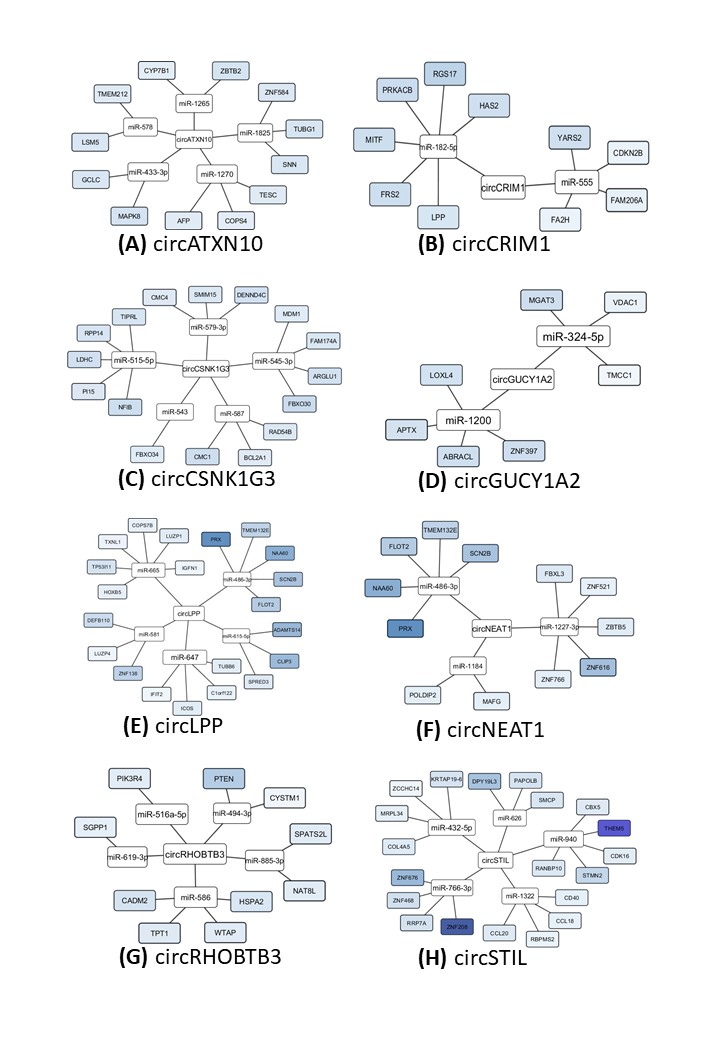


**Supplementary Figure S1.** Bioinformatic analysis of circRNA–miRNA–gene interaction of the eight circRNAs examined in this study. The circRNAs refer to those in Table 2 of the Main text with their characteristcs compiled in the Supplementary Microarray Data File.xlsx and the database circBase [1]. The potential binding sites to miRNAs were computed with the software CircInteractome [2]. MiRNA–gene interactions were identified with the miRDB and TargetScan databases [3,4].

**Supplementary Information S3: RT-qPCR methodology**

***General comments***

RT-qPCR analyses were carried out in accordance to the MIQE guidelines [5] detailed summarized in the following Supplementary Table S1. No-template controls and no-reverse transcriptase controls showed negative results.

**Supplementary Table S1.** MIQE checklist according to Bustin et al. [5].

| **ITEM TO CHECK** | **IMPOR-TANCE** | **CHECK-LIST** | **WHERE; COMMENT** |
| --- | --- | --- | --- |
| **EXPERIMENTAL design** |  |  |  |
| Definition of experimental and control groups | **E** | Yes | Main text: 2. Results, 2.1. Patient characteristics, Table 1. |
| Number within each group | E | Yes | Main text: 2. Results, 2.1. Patient characteristics, Table 1. |
| Assay carried out by core lab or investigator's lab? | D | Yes | Investigator's lab. |
| Acknowledgement of authors' contributions | D | Yes | Section Acknowledgements. |
| **SAMPLE** |  |  |  |
| Description | E | Yes | Main text: 4. Materials and Methods, 4.1. and 4.2. |
| Volume/mass of sample processed | D | Yes | Main text: 4. Materials and Methods, 4.1. and 4.2. |
| Microdissection or macrodissection | E | Yes | Main text: 4. Materials and Methods, 4.1. and 4.2. |
| Processing procedure | E | Yes | Main text: 4. Materials and Methods, 4.1. and 4.2. |
| If frozen - how and how quickly? | E | Yes | Main text: 4. Materials and Methods, 4.1. and 4.2. |
| If fixed - with what, how quickly? | E | Yes | Main text: 4. Materials and Methods, 4.1. and 4.2. |
| Sample storage conditions and duration (esp. for FFPE samples) | E | Yes | Main text: 4. Materials and Methods, 4.1. and 4.2. |
| **Nucleic acid extraction** | |  |  |
| Procedure and/or instrumentation | E | Yes | Main text: 4. Materials and Methods, 4.1. and 4.2. |
| Name of kit and details of any modifications | E | Yes | Main text: 4. Materials and Methods, 4.1. and 4.2. |
| Source of additional reagents used | D | N/A | Not used. |
| Details of DNase or RNase treatment | E | Yes | Main text: 4. Materials and Methods, 4.1. and 4.2.: RNA extraction, on-column DNase digestion. |
| Contamination assessment (DNA or RNA) | E | Yes | Main text: 4. Methods and Materials, 4.2.3. and Supplementary Information S3: General comments: Genomic DNA contamination was excluded by qPCR control experiments without reverse transcription of RNA for all target genes. |
| Nucleic acid quantification | E | Yes | Main text: 4. Materials and Methods, 4.1. and 4.2. |
| Instrument and method | E | Yes | Main text: 4. Materials and Methods, 4.1. and 4.2. |
| Purity (A260/A280) | D | Yes | Main text: 4. Materials and Methods, 4.1. and 4.2. |
| Yield | D | Yes | Main text: 4. Materials and Methods, 4.1. and 4.2. |
| RNA integrity method/instrument | E | Yes | Main text: 4. Materials and Methods, 4.1. and 4.2.: RIN; Bioanalyzer 2100, Agilent RNA 6000 Nano Chip Kit. |
| RIN/RQI or Cq of 3' and 5' transcripts | E | Yes | Main text: 4. Materials and Methods: RIN; Bioanalyzer 2100, Agilent above. |
| Electrophoresis traces | D | Yes | see RNA integrity: Agilent electrophoresis. |
| Inhibition testing (Cq dilutions, spike or other) | E | Yes | Supplementary Information S3: Cq dilution, see characteristics of standard curves in Table S7. |
| **REVERSE transcription** | |  |  |
| Complete reaction conditions | E | Yes | Main text: 4. Materials and Methods, 4.2.3. and Supplementary Information S3: RT-qPCR methodology with Tables S2A and S2B. |
| Amount of RNA and reaction volume | E | Yes | Main text: 4. Materials and Methods, 4.2.3. and Supplementary Information S3: RT-qPCR methodology with Tables S2A and S2B. |
| Priming oligonucleotide (if using GSP) and concentration | E | Yes | Main text: 4. Materials and Methods, 4.2.3. and Supplementary Information S3: RT-qPCR methodology with Tables S2A and S2B. |
| Reverse transcriptase and concentration | E | Yes | Main text: 4. Materials and Methods, 4.2.3 and Supplementary Information S3: RT-qPCR methodology with Tables S2A and S2B. |
| Temperature and time | E | Yes | Main text: 4. Materials and Methods, 4.2.3. and Supplementary Information S3: RT-qPCR methodology with Tables S2A and S2B. |
| Manufacturer of reagents and catalogue numbers | D | Yes | Main text: 4. Materials and Methods, 4.2.3. and Supplementary Information S3: RT-qPCR methodology with Tables S2A and S2B. |
| Cqs with and without RT | D | Yes | Supplementary Information S3: General comments. |
| Storage conditions of cDNA | D | Yes | Main text. 4. Materials and Methods; 4.2.3. and Supplementary Information S3 with cDNA synthesis with storage of samples at ‑20 °C. |
| **qPCR target information** | |  |  |
| Gene symbols | E | Yes | Main text: 2. Results, Table 2; Supplementary Information S3 with Tables S4–S5. |
| If multiplex, efficiency and LOD of each assay. | E | N/A | Only singleplex qPCR. |
| Sequence accession number | E | Yes | Main text: Table 2; Supplementary Information S3: RT-qPCR methodology with Tables S4–S5. |
| Location of amplicon | D | Yes | Supplementary Information S3 with Tables S3-S4. |
| Amplicon length | E | Yes | Supplementary Information S3: Supplementary Tables S3; Bioanalyzer 2100 DNA1000 expert series II Chip analysis: Supplementary Figure S3. |
| In silico specificity screen (BLAST, etc) | E | Yes | Main text: 4. Materials and Methods and Supplementary Information S3: RT-qPCR methodology. All primers and amplicons were checked by screens in different databases, see URL links in "qPCR measurements". |
| Pseudogenes, retropseudogenes or other homologs? | D | N/A |  |
| Sequence alignment | D | Yes | Supplementary Information S3: RT-qPCR methodology, see URL links as mentioned above. Using NCBI-based Megablast against standard Nucleotide collection databases (nr/nt) and RefSeq, filtered Homo sapiens (taxid.9606). Analyses using databases circBase and CircInteractome. |
| Secondary structure analysis of amplicon | D | No |  |
| Location of each primer by exon or intron (if applicable) | E | Yes | Supplementary Information 3: Supplementary Tables S3–S4. Using different databases: Ensembl, NCBI nucleotide, circBase, and CircInteractome. |
| What splice variants are targeted? | E | N/A |  |
| **qPCR oligonucleotides** | |  |  |
| Primer sequences | E | Yes | Supplementary Information 3: RT-qPCR methodology with Supplementary Tables S3 and S5. |
| RTPrimerDB Identification Number | D | N/A |  |
| Probe sequences | D | Yes | Supplementary Information 3: RT-qPCR methodology, Supplementary Table S6, protocols H and I, UPL probes only for ALAS1 and HPRT1. |
| Location and identity of any modifications | E | N/A | No modifications. |
| Manufacturer of oligonucleotides | D | Yes | Main text: 4. Materials and Methods: TIB MolBiol (Berlin, Germany); in kits of ThermoFisher Scientific and Roche. |
| Purification method | D | Yes | TIB MolBiol: GSF purification. |
| **qPCR protocol** |  |  |  |
| Complete reaction conditions | E | Yes | Main text: 4. Materials and Methods, 4.2.3. and Supplementary Information S3: RT-qPCR methodology with Table S6 (Protocols A-I). |
| Reaction volume and amount of cDNA/DNA | E | Yes | Main text: 4. Materials and Methods, 4.2.3. and Supplementary Information S3: RT-qPCR methodology with Table S6 (Protocols of measurement conditions A-I). |
| Primer, (probe), Mg++ and dNTP concentrations | E | Yes | Main text: 4. Materials and Methods, 4.2.3. and Supplementary Information S3: RT-qPCR methodology. |
| Polymerase identity and concentration | E | Yes | Supplementary Information S3: RT-qPCR methodology with Table S6 (Protocol A, H). |
| Buffer/kit identity and manufacturer | E | Yes | Main text: 4. Materials and Methods, 4.2.3. and Supplementary Information S3: RT-qPCR methodology with Table S6 (Protocols of measurement conditions A-I). |
| Exact chemical constitution of the buffer | D | No | The manufacturer does not provide this information. |
| Additives (SYBR Green I, DMSO, etc.) | E | Yes | Main text: 4. Materials and Methods, 4.2.3. and Supplementary Information S3: RT-qPCR methodology with Table S6 (Protocols A-I). SYBR Green in ready-to-use qPCR master (ThermoFisher). |
| Manufacturer of plates/tubes and catalogue number | D | Yes | Main text: 4. Materials and Methods, 4.2.3 and Supplementary Information S3: RT-qPCR methodology with cDNA synthesis and qPCR measurements. |
| Complete thermocycling parameters | E | Yes | Main text: 4. Materials and Methods, 4.2.3 and Supplementary Information S3: RT-qPCR methodology with Table S6 (Protocols A-I). |
| Reaction setup (manual/robotic) | D | Yes | Manual setup. |
| Manufacturer of qPCR instrument | E | Yes | Main text: 4. Materials and Methods, 4.2.3.: LightCycler 480 (Roche). |
| **qPCR VALIDATION** |  |  |  |
| Evidence of optimisation | D | Yes | Supplementary Information S3: RT-qPCR methodology: all run conditions of qPCRs were optimized, see also ref. [6]; for reference genes [7] with primers as indicated in Table S3 and S5; for circ*CSNK1G3* according to Chen et al. [8], for *GUCY1A2* according to Zhang et al. [9]. Supplementary Information S3, Figure S2 and S3 (Melting curves and amplicon size analyses). |
| Specificity (gel, sequence, melt, or digest) | E | Yes | Supplementary Information S3: RT-qPCR methodology with Figure S2 and S3, (Melting curves on the LightCycler and gel views on Agilent Bioanalyzer 2100 electropherograms). Main text: Figure 3A–C. |
| For SYBR Green I, Cq of the NTC | E | Yes | Main text: 4. Materials and Methods, 4.2.3 and Supplementary Information S3: RT-qPCR methodology, General comments. |
| Standard curves with slope and y-intercept | E | Yes | Supplementary Information S3: RT-qPCR methodology with Table S7. |
| PCR efficiency calculated from slope | E | Yes | Supplementary Information S3: RT-qPCR methodology. Performance data with Table S7. |
| Confidence interval for PCR efficiency or standard error | D | Yes | Supplementary Information S3: RT-qPCR methodology, Performance data with Table S7. |
| r2 of standard curve | E | N/A | Not provided by the LC480 software. |
| Linear dynamic range | E | Yes | Supplementary Information S3: RT-qPCR methodology; Performance data with Table S7 with endpoints of standard curves. |
| Cq variation at lower limit | E | Yes | Supplementary Information S3: RT-qPCR methodology, Supplementary Table S7 with Cq range of the measured samples. |
| Confidence intervals throughout range | D | N/A |  |
| Evidence for limit of detection | E | Yes | Supplementary Information S3: RT-qPCR methodology with Supplementary Table S7: Samples with Cq values in the dynamic range, details in the legend. |
| If multiplex, efficiency and LOD of each assay. | E | N/A | No multiplex assays. |
| **DATA ANALYSIS** |  |  |  |
| qPCR analysis program (source, version) | E | Yes | Main text: 4. Materials and Methods, 4.2.3 and Supplementary Information S3: RT-PCR methodology. Performance data with Supplementary Table S7 (LightCycler 480 software, release 1.5.1.62 using the “second derivative maximum” method); qbase^+^ software, version 3.2 (Biogazelle, Zwijnaarde, Belgium) for normalized relative quantities. |
| Cq method determination | E | Yes |  |
| Outlier identification and disposition | E | N/A | See Evidence for limit of detection and Supplementary Table S7. |
| Results of NTCs | E | Yes | Main text: 4. Materials and Methods, 4.2.3 and Supplementary Information S3: RT-PCR methodology, General comments. |
| Justification of number and choice of reference genes | E | Yes | Main text: 4. Materials and Methods, 4.2.3 and Supplementary Information S3 : RT-PCR methodology with two reference genes ALAS1 and HPRT1 according to [7]. |
| Description of normalisation method | E | Yes | Main text: 4. Materials and Methods; Supplementary Information 3: RT-PCR methodology: cancer-specific reference genes ALAS1 and HPRT1 for prostate cancer, see previous comment. Calculations using the software qbase^+^. |
| Number and concordance of biological replicates | D | Yes | Concordance is reflected by the performance data given in the repeatability and reproducibility data in Table 3. |
| Number and stage (RT or qPCR) of technical replicates | E | Yes | Main text: 4. Materials and Methods, 4.2.3 and Supplementary Information 3: RT-PCR methodology, at least technical duplicates in qPCR. |
| Repeatability (intra-assay variation) | E | Yes | Main text: 2. Results with 2.3.2., Table 3. and 4. Materials and Methods, 4.2.3. |
| Reproducibility (inter-assay variation, %CV) | D | Yes | Main text: 2. Results with 2.3.2., Table 3. and 4. Materials and Methods, 4.2.3. |
| Power analysis | D | Yes | Main text: 4. Materials and Methods, 4.3. Statistics and data analysis; Supplementary Information S1; 2. Results: 2.1. Patient characteristics and study design. |
| Statistical methods for result significance | E | Yes | Main text: 4. Materials and Methods: 4.3. Statistics and data analysis. Results, figures, and figure legends. |
| Software (source, version) | E | Yes | Main text: 4. Materials and Methods: 4.3. Statistics and data analysis. Results, figures, and figure legends. |
| Cq or raw data submission using RDML | D | No |  |

E: essential information; D: desirable information if available; N/A: not applicable.

***cDNA synthesis***

Maxima First Strand cDNA Synthesis Kit for RT-qPCR (Thermo Scientific, Waltham, MA, USA; Cat.No. K1642) was used in final reaction volume of 20 µL according to the following protocol:

**Supplementary Table S2A.** cDNA synthesis using the Maxima First Strand cDNA Synthesis Kit.

| Volume (µL) | Reagent/Sample | Components |
| --- | --- | --- |
| 4 | 5x Reaction Mix | Reaction buffer, dNTPs, oligo(dT)_18,_ and random hexamer primers without specified concentrations |
| 2 | Maxima Enzyme Mix | Maxima Reverse Transcriptase (M-MuLV RT) and  Thermo Scientific™ RiboLock™ RNase Inhibitor |
| 2 | Total RNA (500 ng) | Diluted RNA (gDNA free); see RNA isolation |
| 12 | Water, nuclease free |  |

The RT reaction was carried out in 0.2 mL PCR Soft Tubes (Biozym Scientific GmbH, Germany; Article No. 711080) in a thermal block cycler (Biometra GmbH, Göttingen, Germany) as follows: 10 min at 25 °C, followed by 15 min at 50 °C, and terminated by heating at 85 °C for 5 min; end 4 °C. All cDNA samples were stored at -20 °C until qPCR analysis.

As explained in the Main text: 4. Materials and Methods, 4.2.3, we used the Transcriptor First Strand cDNA Synthesis Kit (Life Science Roche, Mannheim, Germany; Cat. No. 04379012001) (Supplementary Table S2B) for the cDNA synthesis of circRNAs for the comparative priming with random hexamer and oligo(dT)_18_ primers according to the following protocol. This procedure was used as one validation method for the circular nature of the examined circRNAs:

**Supplementary Table S2B.** cDNA synthesis using Transcriptor First Strand cDNA Synthesis Kit

| **Volume (µL)** | **Reagent/Sample** | **Components and final (1x) concentration** |
| --- | --- | --- |
| 2 | Total RNA (500 ng) | 1 µg |
| 2  or  1 | Random hexamer primers  or:  Anchored-oligo(dT)_18_ primers | 60 µM  or:  2.5 µM |
| 9 **or** 10 | Water, PCR grade |  |
| 4 | Transcriptor Reverse Transcriptase Reaction Buffer, 5x conc. | 50 mM Tris/HCl, 30 mM KCl, 8 mM MgCl_2_ |
| 0.5 | Protector RNase Inhibitor | 20 U |
| 2 | Deoxynucleotide mix | 1 mM each |
| 0.5 | Transcriptor Reverse Transcriptase | 10 U |

The RT reaction conditions were primer dependent. Using random hexamer primers, the conditions were 10 min at 25 °C, followed by 30 min at 55 °C, and inactivation for 5 min at 85 °C; end 4 °C. Using anchored-oligo(dT)_18_ primers, the initial incubation step was omitted, the other temperature steps were identical. All cDNA samples were stored at -20 °C until qPCR analysis.

***qPCR measurements***

All real-time qPCR runs for the circRNAs and linear RNAs were carried out on the LightCycler 480 Instrument (Roche Molecular Systems, Mannheim, Germany) in white 96-well plates (Cat.No. 04729692001). At least technical duplicates of every cDNA sample were measured and resulting mean Cq values were used for further calculations. Maxima SYBR Green qPCR Master Mix (2x) (Thermo Scientific; Cat.No. K0252) was used. The measurement of the circRNAs is based on their specific backsplice junctions with divergent primers [10]. The backsplice junction sequences of the eight circRNAs and their amplicon characteristics are summarized in Supplementary Table S3 and confirmed by Sanger sequencing of qPCR products (Figure 3), while information concerning the linear counterparts of the circRNAs and the normalizers are listed in Supplementary Table S4.

The Primer3 (http://bioinfo.ut.ee/primer3/) and the Roche/UPL ProbeFinder web-based softwares (<https://lifescience.roche.com/en_de/brands/universal-probe-library.html#assay-design-center>) were used to design all primers of circRNAs (divergent) and mRNAs (convergent) (Supplementary Table S5) [11]. Genes and primer sequences were checked using the following database links: https://circinteractome.nia.nih.gov/; http://www.circbase.org/; http://www.ensembl.org (Ensembl release 99 - January 2020); https://www.ncbi.nlm.nih.gov/nucleotide; https://blast.ncbi.nlm.nih.gov/Blast.cgi; <https://www.ncbi.nlm.nih.gov/tools/primer-blast/>. Primers were synthesized by TIB Molbiol GmbH (Berlin, Germany).

Protocols for qPCR measurements are summarized in Supplementary Table S6 (Protocols A–I).

Quantitative PCR data analysis was done using qbase^+^ software, version 3.2 (Biogazelle, Zwijnaarde, Belgium; [www.qbaseplus.com](http://www.qbaseplus.com)).

**Supplementary Table S3**. Sequences of amplicons of circRNAs with marked backsplice junctions, primer sequences, and their locations in the host gene

| **CircRNA**  **(name in manuscript) ^a^** | **Amplicon Sequences and -lengths with Backsplice Junction (3'...........5') ^b^** | **Divergent Primers**  **(5'......... 3')** | **Primer Locations**  **(Exon no.) ^c^** |
| --- | --- | --- | --- |
| circ*ATXN10* | TGTGGCCAATGGGTTTAAGTCTCATCTCATTCGTCTGATTGGAAATCTGTGTTACAAGAATAAAGATAACCAA**GACAAGCTTTTC**GCTGTGGCCTGCAGTTTTTAGGCAACATTGCCTCACGGAATGAAGATTCCC **(136 bp)** | Forward:TGTGGCCAATGGGTTTAAGT  Reverse:GGGAATCTTCATTCCGTGAG | Ex9 (F)/Ex4 (R) |
| circ*CRIM1* | CCCGGACAGCTATGAAACTCAAGTCAGACTAACTGCAGATGGTTGCTGTACTTTGCC**AACAAGAAGAGA**AGCCAGATTGCTCCAAGGCCCGCTGTGAAGTCCAGTTCTCTCCACGTTGTCCTGA **(124 bp)** | Forward:CCCGGACAGCTATGAAACTC  Reverse:TCAGGACAACGTGGAGAGAAC | Ex4 (F)/Ex3 (R) |
| circ*CSNK1G3* | GCACCACAGCTACATTTGGAATACAGATTCTATAAGCAGTTAGGAT**CTGGAGCTCTCT**ATCAATATCAGCTCACATCATTGAAAAGATAATTTTGAAGACATGTTTTGCTGAAAAGACACTAAGAAAAATTTTACGAATGGGATGAACATGCTCC **(155 bp)** | Forward:GCACCACAGCTACATTTGGA  Reverse:GGAGCATGTTCATCCCATTC | Ex3 (F)/Ex1 (R) |
| circ*GUCY1A2* | GCTCCTATGCAGACCACTCCAACAAAGAAGAAATTGAAGATGTCTCAGGAATTCTTCAGTGTACTGCTAATA**TACTCGCCTCAG**ACGATACAGCAGACTCTCAAGAGGACACTGCAGTATTATGAACATCAAGTTATTGGTTACAGGGATGCAGAAA **(157 bp)** | Forward:GCTCCTATGCAGACCACTCC  Reverse:TTTCTGCATCCCTGTAACCA | Ex3 (F)/Ex3-2 (R) |
| circ*LPP* | ATTGCAG**CCAAAGAGCTCC**ACTGGTTCAACAGCCTCTCCTCCAGTTTCGACCCCAGTCACAGGACACAAGAGAATGGTCATCCCGAACCAACCCCCTCTAACAGCAACC **(109 bp)** | Forward:ATTGCAG**CCAAAGAGCTCC**AC  Reverse:GGTTGCTGTTAGAGGGGGTT | Ex6 (F, junction- spanning)/Ex6 (R) |
| circ*NEAT1* | GTGTGTTTGGAG**GCTGAATGCAGT**GGTTCCTGGAAGCCAGTGGCACGTTTCCCCGCGTAGCTCGCTTATCCCACAGC **(77 bp)** | Forward:GTGTGTTTGGAG**GCTGAATG**  Reverse:GCTGTGGGATAAGCGAGCTA | Intronless lncRNA  (F, junction- spanning)/(R) |
| circ*RHOBTB3* | TTCTGGGGATGTTTCAAATGTAATCGAGAAAGTTAAATGCATTTTAAAAACACCA**GGAAAGAAAAAA**TGCCTGTCTTAAAGGCTGAAGCGTCACATTATAACTCTGACTTAAATAACTTGCTGTTCTGCTGCCAGTGTGT **(140 bp)** | Forward:TTCTGGGGATGTTTCAAATG  Reverse:ACACACTGGCAGCAGAACAG | Ex7 (F)/Ex6 (R) |
| circ*STIL* | TGATGGGACTATCTCCAGATGCATATCGGTTCCTCACAGAACAAGACAGACAGCTAAGACTACTTCAG**GCACAGGTCTTC**CCA **(83 bp)** | Forward:TGATGGGACTATCTCCAGATGC  Reverse:TGG**GAAGACCTGTGC**CTGAA | Ex12 (F)/  Ex12-11 (R, junction- spanning) |

^a^ Name of the circRNA used in this manuscript; the official name and symbols of circRNAs and host genes see Table 2 in the Main text and and the following Supplementary Table S4 and S5. ^b^ Numbers in brackets indicate the length of amplicons. ^c^ Exon no. according to NCBI RefSeq. Accession no. of the host genes in Table 2 of the Main text and the following Supplementary Table S4.

**Supplementary Table S4.** QPCR target information of the linear RNAs and the reference genes

| **RNA Name in the Manuscript** | **Official Symbol and Full Name of the Host Gene** | **NCBI RefSeq. Accession no.** | **Primer Location**  **(Exon no. in RefSeq.)** | **Intron Spanning size (nt)** |
| --- | --- | --- | --- | --- |
| lin*ATXN10* | *ATXN10*  (Ataxin 10, transcript variant 1) | NM_013236.4 | Ex9 (F)/  Ex10 (R) | 66420 |
| lin*CRIM1* | *CRIM1*  (Cysteine rich transmembrane BMP regulator 1) | NM_016441.3 | Ex6 (F)/  Ex7 (R) | 2425 |
| lin*CSNK1G3* | *CSNK1G3*  (Casein kinase 1 gamma 3, transcript variant 4) | NM_001044723.2 | Ex6 (F)/  Ex8 (R) | 273/1901 |
| lin*GUCY1A2* | *GUCY1A2*  (Guanylate cyclase 1 soluble subunit alpha 2, transcript variant 2) | NM_000855.3 | Ex7 (F+R) | - |
| lin*LPP* | *LPP*  (LIM domain containing preferred translocation partner in lipoma, transcript variant 1) | NM_005578.5 | Ex5 (F+R) | - |
| linNEAT | *NEAT1*  (Nuclear paraspeckle assembly transcript 1, transcript variant MENbeta, long non-coding RNA) | NR_131012.1 | lncRNA | - |
| lin*RHOBTB3* | *RHOBTB3*  (Rho related BTB domain containing 3) | NM_014899.4 | Ex5 (F)/Ex6 (R) | 3045 |
| lin*STIL* | *STIL*  (STIL centriolar assembly protein, transcript variant 3) | NM_001282936.1 | Ex17+18(F)/  Ex18 (R) | 8366 |
| *ALAS1* | *ALAS1*  (5'-aminolevulinate synthase) | NM_000688.6 | Ex4 (F)/  Ex5 (R) | 1128 |
| *HPRT1* | *HPRT1*  (Hypoxanthine phosphoribosyl-transferase 1) | NM_000194.3 | Ex3 (F)/  Ex4+5 (R) | 11100/  3657 |

**Supplementary Table S5.** List of Primer sequences of circRNAs, linear RNAs, and normalizers

| **CircRNAs (circBase ID)** | **Divergent Primer Sequences (5’… 3’)** |
| --- | --- |
| circ*ATXN10*  (*hsa*_circ_0001246) | F: TGTGGCCAATGGGTTTAAGT |
|  | R: GGGAATCTTCATTCCGTGAG |
| circ*CRIM1*  (*hsa*_circ_0007386) | F: CCCGGACAGCTATGAAACTC |
|  | R: TCAGGACAACGTGGAGAGAAC |
| circ*CSNK1G3*  (*hsa*_circ_0001522) ^a^ | F: GCACCACAGCTACATTTGGA |
|  | R: GGAGCATGTTCATCCCATTC |
| circ*GUCY1A2*  (*hsa*_circ_0008602) ^b^ | F: GCTCCTATGCAGACCACTCC |
|  | R: TTTCTGCATCCCTGTAACCA |
| circ*LPP*  (*hsa*_circ_0003759) | F: ATTGCAGCCAAAGAGCTCCAC |
|  | R: GGTTGCTGTTAGAGGGGGTT |
| circ*NEAT1*  (*hsa*_circ_0000324) | F: GTGTGTTTGGAGGCTGAATG |
|  | R: GCTGTGGGATAAGCGAGCTA |
| circ*RHOBTB3*  (*hsa*_circ_0007444) | F: TTCTGGGGATGTTTCAAATG |
|  | R: ACACACTGGCAGCAGAACAG |
| circ*STIL*  (*hsa*_circ_0000069) | F: TGATGGGACTATCTCCAGATGC |
|  | R: TGGGAAGACCTGTGCCTGAA |
| **Linear Transcripts**  **(NCBI mRNA RefSeq Acc. no)** | **Convergent Primer Sequences (5’… 3’)** |
| lin*ATXN10*  (NM_013236.4) | F: ACAAGAATAAAGATAACCAAGACAAGG |
|  | R: TCACTGATGTTGCAGTTGTCC |
| lin*CRIM1*  (NM_016441.3) | F: CTACGTGCCCGAAGGAGA |
|  | R: CAGCCAGCGGGATTATTAAA |
| lin*CSNK1G3*  (NM_001044723.2) | F: TGAGAGGCAGTCTTCCTTGG |
|  | R: ACATAACGAAGATATGTTGCCATT |
| lin*GUCY1A2*  (NM_000855.3) | F: CGTGCTGGCTGGAGTTGTTG |
|  | R: TGGTAAGTGGTTGGGCTGACAT |
| lin*LPP*  (NM_005578.5) | F: GGAGGCAAGACACTTGAGGA |
|  | R: AGATGCTGGTCAAGGAGTCAA |
| lin*NEAT1*  (NR_131012.1) | F: GCAGGTTGGGACTTAGATGG |
|  | R: ACTATGGTGCGGGCACTTAC |
| lin*RHOBTB3*  (NM_014899.4) | F: CCACCTCAACTTGAACAACCA |
|  | R: GGCAGCAGAACAGCAAGTTA |
| lin*STIL*  (NM_001282936.1) | F: ATAACGTGGATCACGCCAGT |
|  | R: CATGCAGTTTAATCCAGAGATCA |
| **Normalizers** |  |
| *ALAS1*  (NM_000688.5) ^c^ | F: GAAATGAATGCCGTGAGGAA |
|  | F: CCTCCATCGGTTTTCACACT |
| *HPRT1*  (NM_000194) ^d^ | F: TGATAGATCCATTCCTATGACTGTAGA |
|  | R: AAGACATTCTTTCCAGTTAAAGTTGAG |

Abbreviations: F, forward primer; R, reverse primer.

^a^ According to Chen et al. [8]. ^b^ According to Zhang et al. [9]. ^c^ With Roche UPL Probe #40 see Supplementary Table S6, protocols H, I. ^d^ With Roche UPL Probe #22, see Supplementary Table S6, protocols H, I.

**Supplementary Table S6.** Protocols for LightCycler 480 qPCR runs (SYBR Green or Probe assay format)

In the following protocols A-I, the measurement conditions for all 20 analytes (8 circRNAs, 8 linRNAs, and the 2 normalizers) are compiled. In order to simplify the overview, we have combined the respective tests with the same reaction conditions. Assays based on SYBR Green (protocols A-G) and hydrolysis probes (protocols H, I) were applied.

**A)** qPCR reaction mix for all SYBR Green assays

| **Volume (µL)** | **Reagent/Sample** | **Components** |
| --- | --- | --- |
| 5 | Maxima SYBR Green qPCR Master Mix (2X) | Maxima Hot Start Taq DNA Polymerase, dNTPs (also dUTP) and SYBR Green I in an optimized PCR buffer |
| 2 | Primer Mix | Forward and reverse primer mix, final concentration 0.5 µM |
| 2 | Water | nuclease free |
| 1 | cDNA | undiluted |
| **Total volume 10 µL** | |  |

**B)** General template for LightCycler 480 SYBR Green assay runs

| **Setup** | | | |
| --- | --- | --- | --- |
| Block type | | Reaction volume (µL) | |
| 96 | | 10 | |
| Detection format | Excitation filter |  | Emission filter |
| SYBR Green | 483 nm | | 533 nm |
| **Programs** | | | |
| Program names | Cycles | | Analysis mode |
| Pre-incubation | 1 | | None |
| Amplification | 45 | | Quantification |
| Melting curve | 1 | | Melting curve |
| Cooling | 1 | | None |
|  |  | |  |

**C)** Setup and program steps of pre-incubation, melting curve analysis, and cooling step were identical for all assays in SYBR Green detection format as follows

| **Temperature targets** | | | | | |
| --- | --- | --- | --- | --- | --- |
|  | Target (°C) | Acquisition mode | Hold time (s) | Ramp rate (°C/s) | Acquisitions (per °C) |
| Pre-incubation | 95 | None | 600 | 4.4 | - |
| Amplification |  | Gene dependent steps | | | |
| Melting curve | 95 | None | 5 | 4.4 | - |
|  | 65 | None | 60 | 2.2 | - |
|  | 95 | Continuous | - | 0.11 | 5 |
| Cooling | 40 | None | 30 | 2.2 | - |

**D)** Amplification run template for all linear RNAs and circ*ATXN10*, circ*LPP*, circ*RHOBTB3*, and circ*STIL*

| **Temperature targets** | | | | | |
| --- | --- | --- | --- | --- | --- |
|  | Target (°C) | Acquisition mode | Hold time (s) | Ramp rate (°C/s) | Acquisitions (per °C) |
| Amplification | 95 | None | 15 | 4.4 | - |
|  | 60 | None | 15 | 1.0 | - |
|  | 70 | Single | 15 | 4.4 | - |

**E)** Amplification run template for circ*CSNK1G3* and circ*GUCY1A2*

| **Temperature targets** | | | | | |
| --- | --- | --- | --- | --- | --- |
|  | Target (°C) | Acquisition mode | Hold time (s) | Ramp rate (°C/s) | Acquisitions (per °C) |
| Amplification | 95 | None | 10 | 4.4 | - |
|  | 60 | None | 30 | 2.2 | - |
|  | 72 | Single | 2 | 4.4 | - |

**F)** Amplification run template for circ*CRIM1*

| **Temperature targets** | | | | | |
| --- | --- | --- | --- | --- | --- |
|  | Target (°C) | Acquisition mode | Hold time (s) | Ramp rate (°C/s) | Acquisitions (per °C) |
| Amplification | 95 | None | 15 | 4.4 | - |
|  | 60 | None | 30 | 2.2 | - |
|  | 79 | Single | 2 | 4.4 | - |

**G)** Amplification run template for circ*NEAT1*

| **Temperature targets** | | | | | |
| --- | --- | --- | --- | --- | --- |
|  | Target (°C) | Acquisition mode | Hold time (s) | Ramp rate (°C/s) | Acquisitions (per °C) |
| Amplification | 95 | None | 15 | 4.4 | - |
|  | 60 | None | 15 | 1.0 | - |
|  | 70 | None | 15 | 4.4 | - |
|  | 79 | Single | 2 | 4.4 | - |

**H)** qPCR reaction mix for hydrolysis probe assays for the reference genes *ALAS1* and *HPRT1*

| **Volume (µL)** | **Reagent/Sample** | **Components** |
| --- | --- | --- |
| 5 | 2x LightCycler Probes Master | Ready-use-hot start PCR mix: FastStart Taq DNA Polymerase, reaction buffer, dNTPs (with dUTP) and 6.4 mM MgCl_2_ (Cat.no. 04707494001) |
| 1 | Primer Mix | Forward and reverse primer mix, final concentration 0.25 µM |
| 1 | Probe | Roche UPL Probe #40 for ALAS1 or #22 for HPRT1, final concentration 0.2 µM |
| 2 | Water | nuclease free |
| 1 | cDNA | Undiluted |
| **Total volume 10 µL** | |  |

**I)** Setup and program steps for the hydrolysis probe-based assays of *ALAS1* and *HPRT1*

| **Setup** | | | | | |
| --- | --- | --- | --- | --- | --- |
| Block type | | | Reaction Volume (µL) | | |
| 96 | | | 10 | | |
| Detection format | | Excitation filter |  | Emission filter | |
| Mono Color Hydrolysis Probe | | 483 nm | | 533 nm | |
| **Programs** | | | | | |
| Program names | | Cycles | | Analysis mode | |
| Pre-incubation | | 1 | | None | |
| Amplification | | 45 | | Quantification | |
| Cooling | | 1 | | None | |
| **Temperature targets** | | | | | |
|  | Target (°C) | Acquisition mode | Hold time (s) | Ramp rate (°C/s) | Acquisitions (per °C) |
| Pre-incubation | 95 | None | 600 | 4.4 |  |
| Amplification | 95 | None | 10 | 4.4 |  |
|  | 60 | None | 30 | 2.2 |  |
|  | 72 | Single | 1 | 4.4 |  |
| Cooling | 40 | None | 60 | 1.0 |  |

*Performance data of RT-qPCR analyses*

In the following, the analytical specificity of the RT-PCR products in all assays are summarized in Figure S2, Figure S3, and Table S7.

| **Gene name** | **Tm-curve of circular PCR product** | **Tm-curve of linear PCR product** |
| --- | --- | --- |
| *ATXN10*  Circ: Tm-peak 79 °C  Lin: Tm-peak 76 °C | 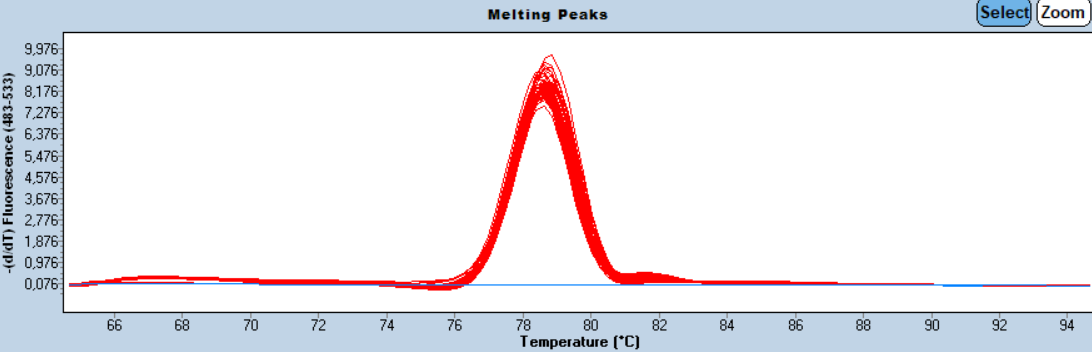 | 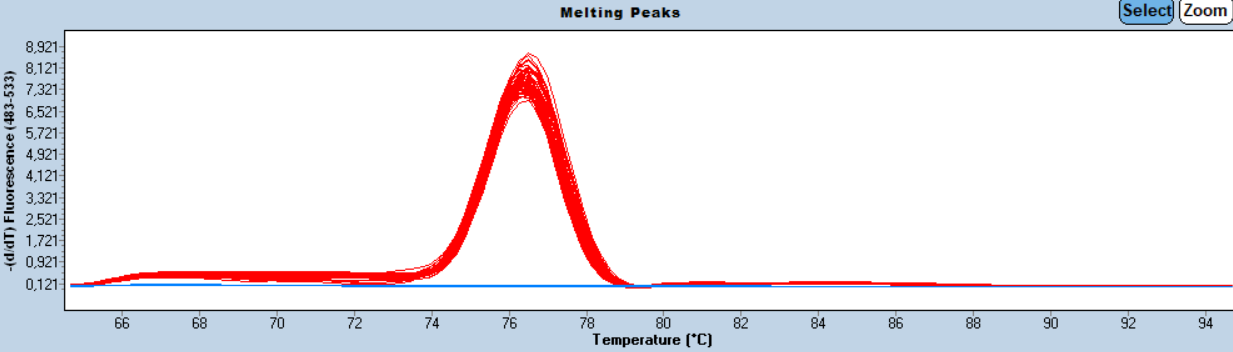 |
| *CRIM1*  Circ: Tm-peak 81 °C  Lin: Tm-peak 79 °C | 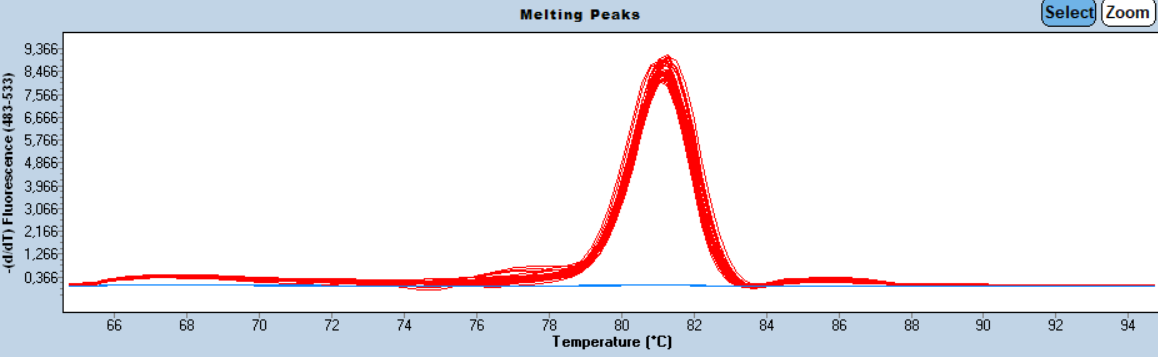 | 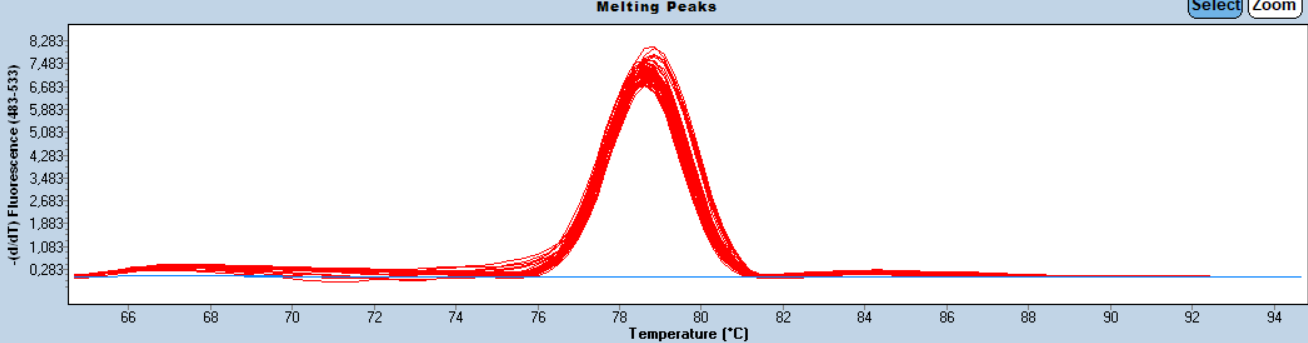 |
| *CSNK1G3*  Circ: Tm-peak 76 °C  Lin: Tm-peak 78 °C | 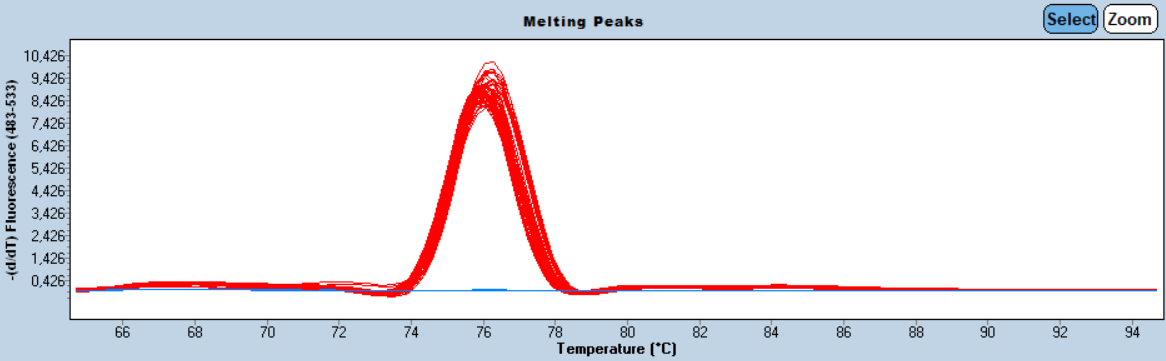 | 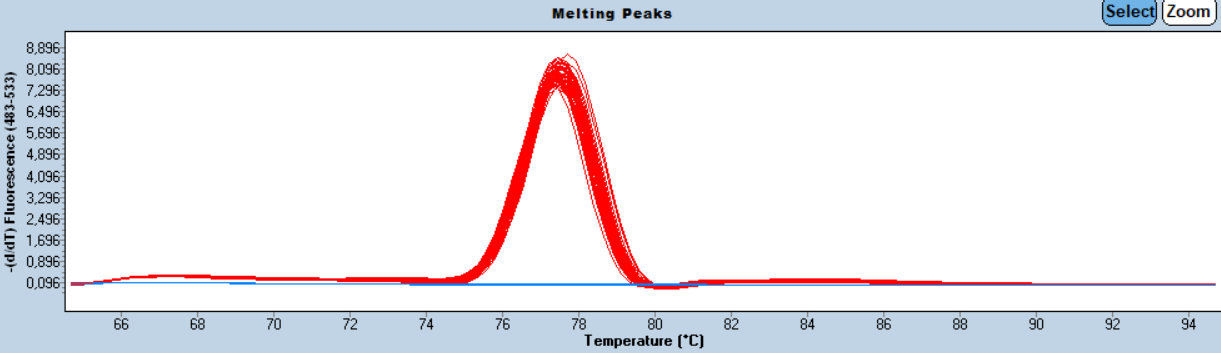 |
| *GUCY1A2*  Circ: Tm-peak 78 °C  Lin: Tm-peak 82 °C | 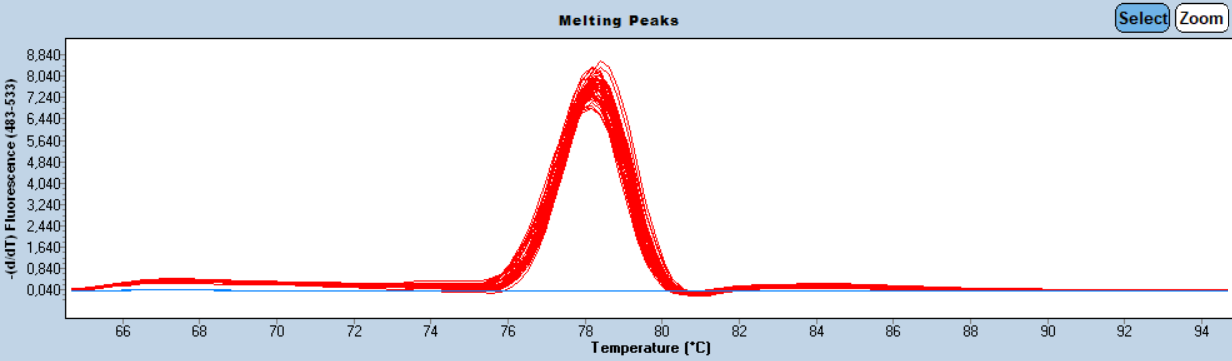 | 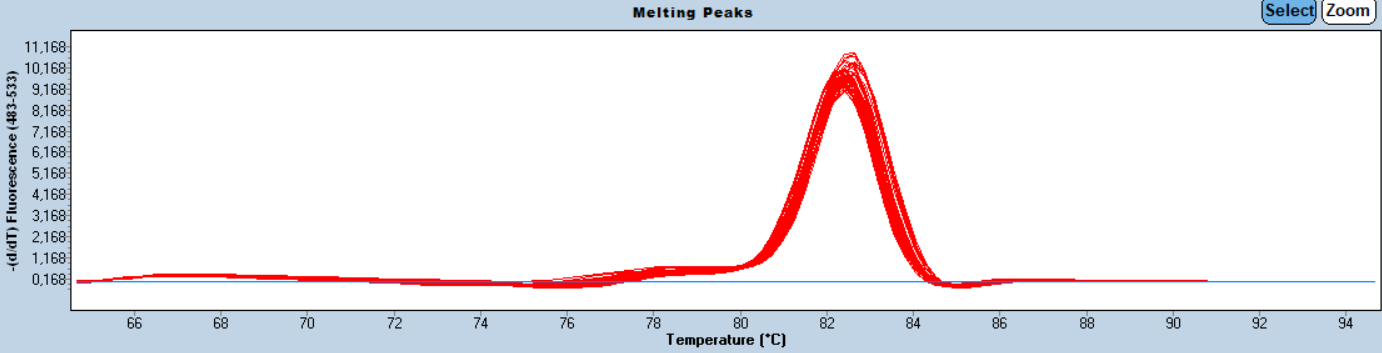 |
| *LPP*  Circ: Tm-peak 82 °C  Lin: Tm-peak 81 °C | 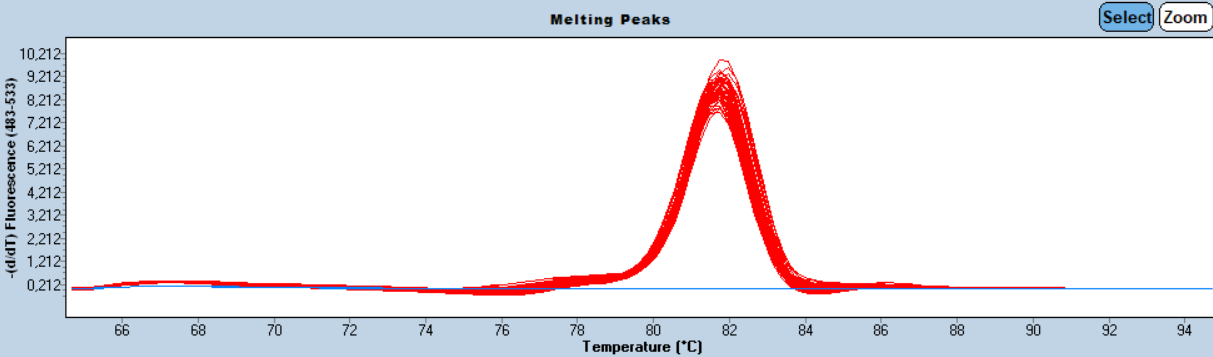 | 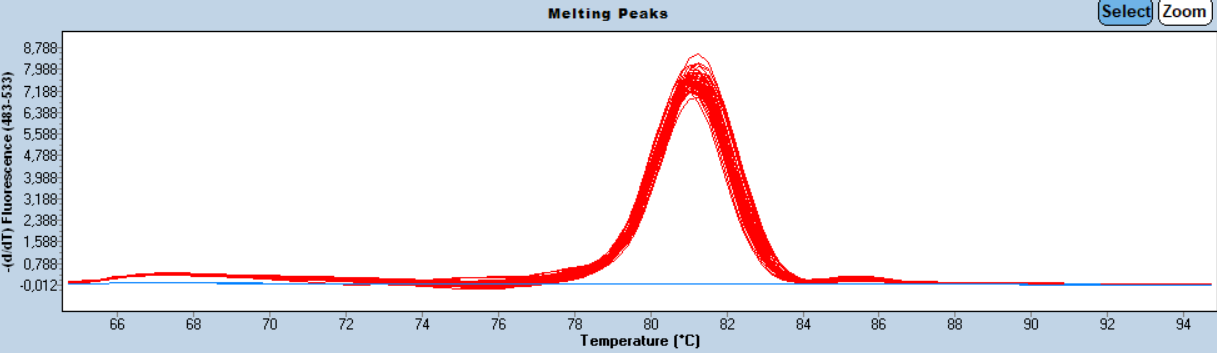 |
| *NEAT1*  Circ: Tm-peak 82 °C  Lin: Tm-peak 80 °C | 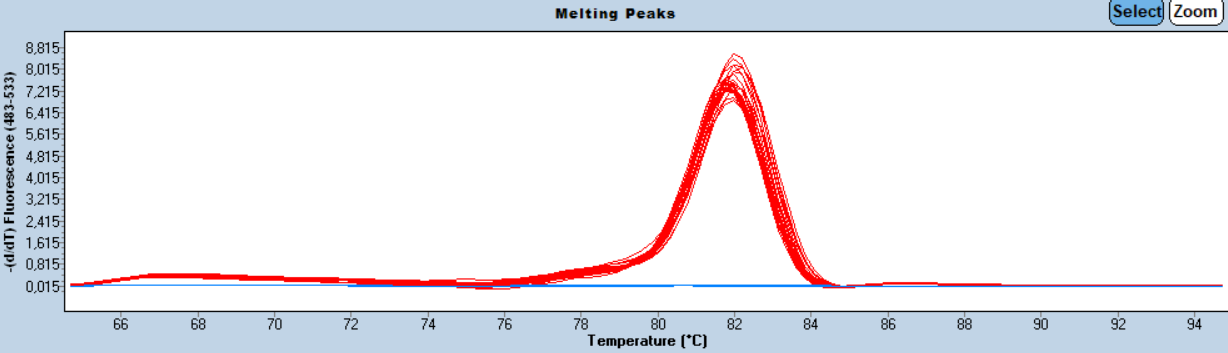 | **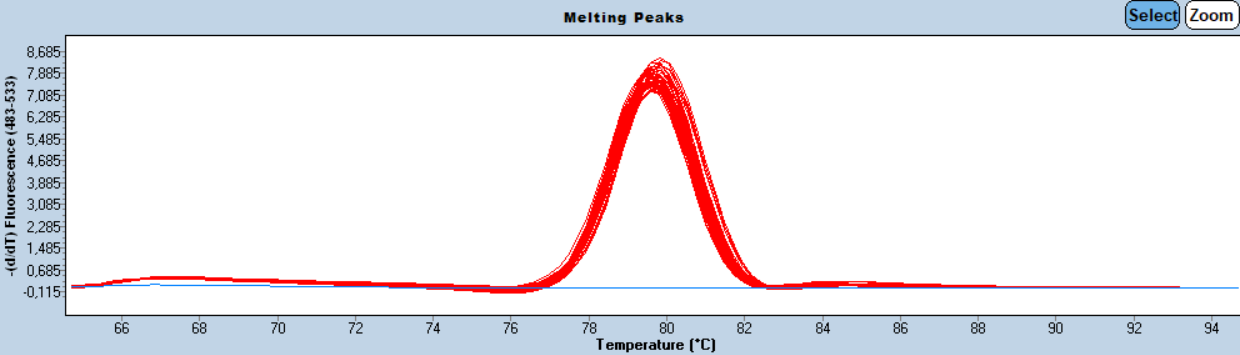** |
| *RHOBTB3*  Circ: Tm-peak 77 °C  Lin: Tm-peak 77 °C | 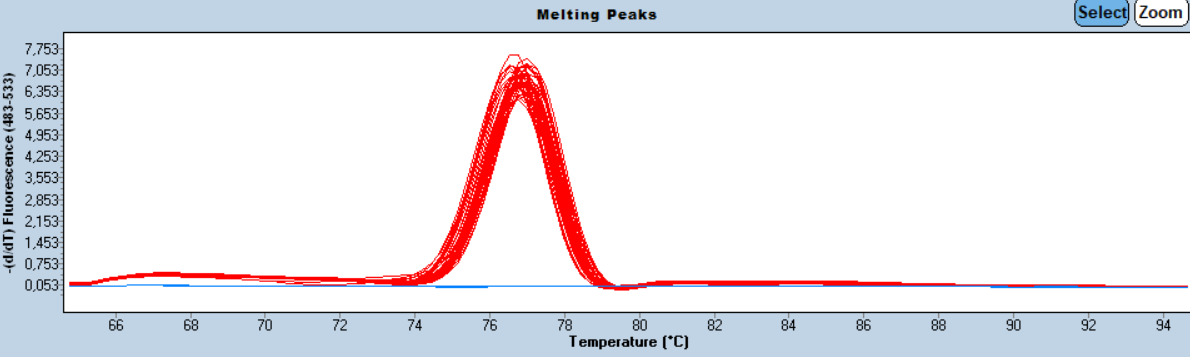 | 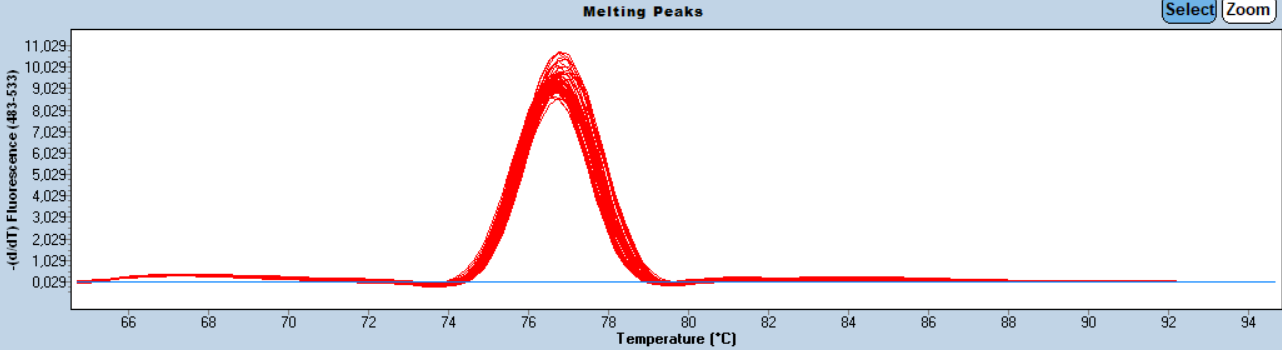 |
| *STIL*  Circ: Tm-peak 78 °C  Lin: Tm-peak 79 °C | 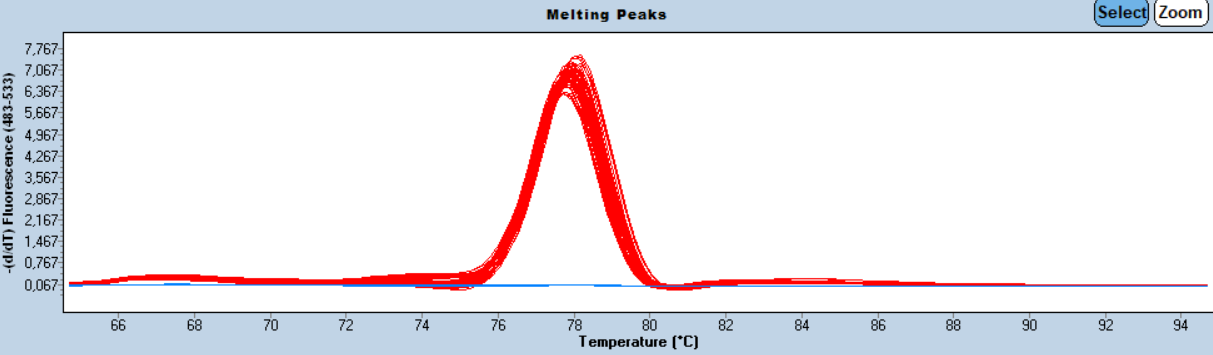 | 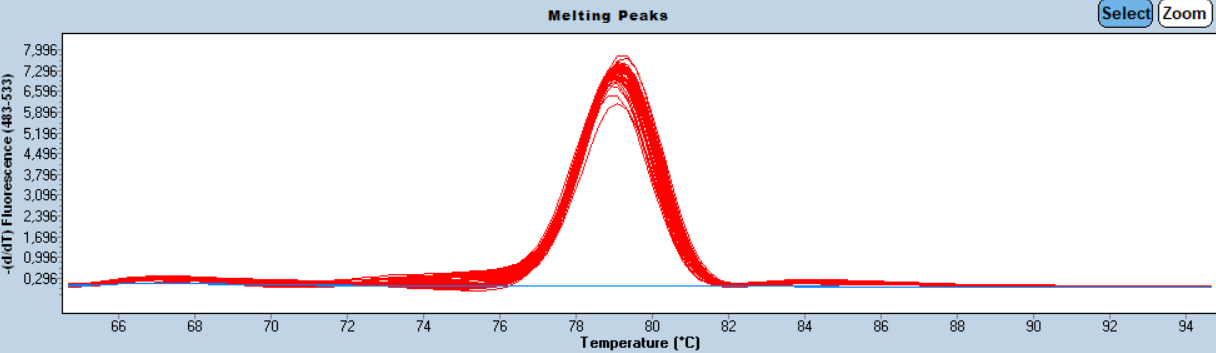 |

**Supplementary Figure S2:** Melting curve analysis of circular and linear qPCR products on LightCycler 480. Abbreviation: Tm, melting temperature.

**
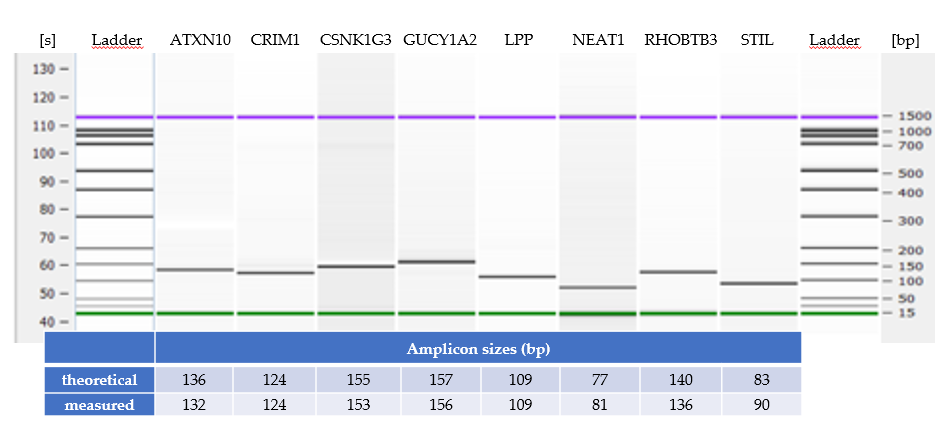
(A) Circular RNAs**


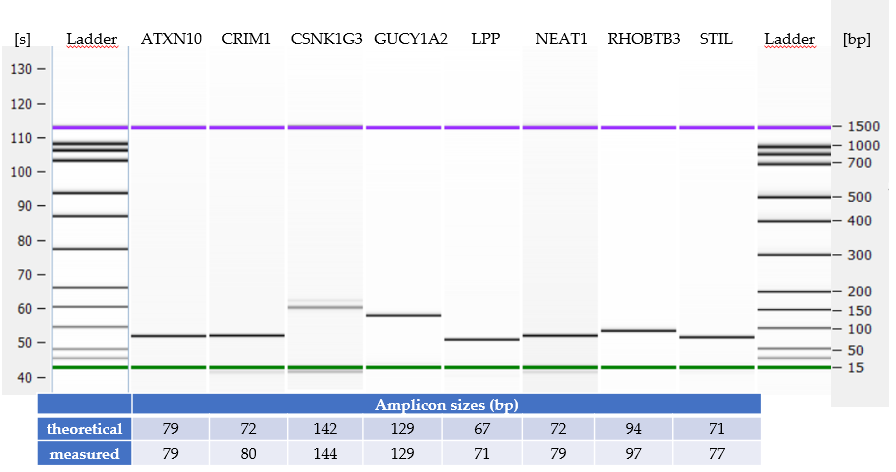
**(B) Linear RNAs**

**Supplementary Figure S3.** Gel views of analyzed amplicons of (A) cirRNAs and (B) linear RNAs with Bioanalyzer 2100 (comparison files). The determined amplicon sizes (bp) are within the size accuracy range of about 10% according to Agilent Bioanalyzer 2100 specifications. For linear *CSNK1G3*, the primer pair of Chen et al. was used that detects alternatively spliced transcript variants encoding multiple isoforms of this gene with theoretical products lengths of 142 and 145 bp (NCBI Primer Blast-RefSeq search by primer sequences input).

**Supplementary Table S7.** Characteristics of the standard curves of qPCR analyses. Diluted cDNAs or diluted amplicons were used to generate standard curves. The LightCycler 480 Software Version 1.5.1.62 using the "second derivative maximum" method was applied to calculate Cq values. All the characteristics (see headline of the Table) were calculated by the LightCycler software).

| **RNA** | **PCR efficiency ^a^** | **Slope** | **y-Intercept** | **Error ^b^** | **Dynamic range ^c^** | **Cq range of samples ^d^** |
| --- | --- | --- | --- | --- | --- | --- |
| **circ*ATXN10*** | 1.908 | -3.564 | 10.21 | 0.0064 | 10.21-34.73 | 21.72-28.59 |
| **circ*CRIM1*** | 1.947 | -3.457 | 9.883 | 0.0283 | 9.88-34.12 | 21.55-29.06 |
| **circ*CSNK1G3*** | 1.948 | -3.453 | 20.79 | 0.0121 | 20.49-31.47 | 19.73-25.88 ^e^ |
| **circ*GUCY1A2*** | 1.993 | -3.328 | 21.57 | 0.0201 | 21.70-31.56 | 21.27-28.95 ^e^ |
| **circ*LPP*** | 1.929 | -3.505 | 8.964 | 0.0070 | 10.02-33.59 | 23.07-27.98 |
| **circ*NEAT1*** | 1.991 | -3.43 | 14.64 | 0.0115 | 14.63-35.31 | 32.50-39.88 ^f^ |
| **circ*RHOBTB3*** | 2.041 | -3.228 | 20.35 | 0.0189 | 21.48-33.29 | 22.66-28.88 |
| **circ*STIL*** | 1.935 | -3.487 | 26.26 | 0.0152 | 26.16-34.07 | 26.27-32.36 |
| **lin*ATXN10*** | 2.052 | -3.203 | 18.84 | 0.0042 | 18.79-33.69 | 18.81-24.33 |
| **lin*CRIM1*** | 2.054 | -3.198 | 19.58 | 0.0121 | 19.62-33.94 | 19.58-25.07 |
| **lin*CSNK1G3*** | 1.949 | -3.451 | 20.77 | 0.0112 | 20.49-31.47 | 20.30-26.30 |
| **lin*GUCY1A2*** | 1.976 | -3,382 | 20.53 | 0.0359 | 20.52-34.18 | 20.87-28.01 |
| **lin*LPP*** | 1.941 | -3.473 | 14.19 | 0.0134 | 14.19-34.12 | 16.74-22.06 |
| **lin*NEAT1*** | 2.011 | -3.296 | 13.22 | 0.0041 | 13.17-33.76 | 15.66-21.38 |
| **lin*RHOBTB3*** | 1.929 | -3.504 | 13.50 | 0.0134 | 13.48-32.27 | 19.54-24.18 |
| **lin*STIL*** | 1.954 | -3.437 | 21.23 | 0.0066 | 21.21-34.74 | 22.36-29.66 |
| ***ALAS1*** | 1.920 | -3.529 | 18.89 | 0.0118 | 18.89-34.18 | 21.08-26.68 |
| ***HPRT1*** | 1.863 | -3.702 | 8.881 | 0.0100 | 9.01-31.34 | 23.82-29.95 |

^a^ The PCR-efficiency is calculated by the LightCycler 480 software according to the formula "efficiency=10^-1/slope^.

^b^ The error value is the mean squared error of the single data points fit to the regression line according to the LightCycler 480 operator’s manual. ^c^ Dynamic range represents the range of mean Cq values between the highest and the lowest Cq values of the generated standard curve. ^d^ Cq range of the measured samples represents the lowest and highest Cq values that were measured in samples of this study. ^e^ Cq values of five circ*CSNK1G3* samples and one circ*GUCY1A2* sample, corresponding to 2.6% and 0.5% of all 194 samples examined, were below the Cq values of the dynamic range of the standard curves, but were accordingly extrapolated in the data analysis with qbase^+^. ^f^ Only 145 out of 194 samples (~75%) showed evaluable amplification and melting curves with Cq values within the dynamic range of the standard curve and the upper limit of the reproducibility control sample (mean Cq + 2 SD: 36.80 + 0.309 = 37.42). Normalized relative quantities were calculated only for these samples in qbase^+^.

**Supplementary Information S4: circRNA validation methods**

As explained in Methods and Materials, validation methods were performed to confirm the circular nature of the circRNAs. These methods are based on the detailed description in our previous report [6] and are here briefly summarized.

***RNase R digestion of isolated total RNA***

The exonuclease RNase R digests linear RNAs, while circRNAs are generally resistant to RNase R [12-14]. Although exceptions have been described for circRNAs and linear RNAs with regard to this property of RNase R [15-17], the detection of resistance to RNase R serves as an essential proof of the circular nature of circRNAs. We performed RNase R treatment with total RNA extracted from pooled PCa tissue samples. To 1 µg of total RNA, 3 U of RNase R (Epicentre, Illumina, San Diego, USA), 10 U of Ribonuclease Inhibitor (abm, Richmond, Canada), reaction buffer, and water in a total volume of 10 µL were added. This mixture was incubated at 37°C for 15 minutes [12]. Control samples contained water instead of RNase R. After incubation, QIAzol lysis reagent was added and total RNA isolation was performed using the RNeasy Mini Kit (Qiagen) as described in Section 4 of the Main text. RT-qPCR measurements were performed for all circRNAs and linear RNAs of this study (see Supplementary Information S3). The 2^-ΔΔCT^ approach was used to calculate fold change between expressions of RNase R treated and control treated samples as described previously based on the normalization to the C. elegans spike-in [6,14] (see Main text, Results 2.3.1., Figure 3A).

***Random hexamer primers vs. oligo(dT)_18_ primers for cDNA synthesis***

When using random hexamer primers in comparison to oligo(dT)_18_ primers for cDNA synthesis of circRNA, distinct reduced quantification cycle (Cq) values in qPCR measurements are characteristic as the covalently closed structures circRNAs lack a poly-A-tail to bind oligo(dT) primers for cDNA synthesis [18]. These comparative measurements were performed with a pool of total RNA extracted from PCa tissue samples and cDNA synthesis with the Roche Transcriptor First Strand cDNA Synthesis Kit (Roche, Mannheim, Germany) using random hexamer primers (Roche, Mannheim, Germany) in comparison to Anchored oligo(dT)_18_ primers (Roche, Mannheim, Germany) (see cDNA synthesis mentioned above in Supplementary Information S3 and in Results 2.3.1., Figure 3B).

***Sanger-Sequencing of circRNA backsplice junction***

The backsplice junction sequence for every circRNA is formed by the connection of a downstream 3’ end and an upstream 5’ end. Sequencing of the backsplice junctions can identify circRNAs. For that purpose, RT-qPCR amplifications of all circRNAs examined in this study were performed with divergent primers as indicated in Supplementary Table S5. The corresponding PCR products were detected by agarose gel electrophoresis, eluted, and purified for Sanger sequencing (LGC Genomics GmbH, Berlin, Germany). Backsplice junction sequences (Supplementary Table S3) were validated by Sanger sequencing using the same primers as for qPCR. Circ*LPP*, circ*NEAT1*, and circ*STIL* were only sequenced in one direction as one of the primers was junction spanning (Supplementary Table S3). Sequencing results are presented in Figure 3C of the Main text. The sequencing result of circ*RHOBTB3* was the same as that in kidney carcinoma [6].

**Supplementary Information S5: Associations between circRNAs, linear transcripts, and clinicopathological variables**

**Supplementary Table S8.** Expression ratios of circular and linear RNAs in 79 paired tumor tissue to adjacent normal tissue samples from prostate carcinoma tissue specimens. ^a^

| **RNA** | **Regulation** | **Expression Ratio of RNAs in Tumor to Normal Tissue** | | | |
| --- | --- | --- | --- | --- | --- |
|  |  | **Median (95% CI)** | **Interquartile Range** | ***p-V*alue ^b^** | |
|  |  | *Circular RNAs* |  |  |  |
| circ*ATXN10* | down | 1.74 (1.51 - 2.11) | 1.10 - 3.00 | < 0.0001 |  |
| circ*CRIM1* | down | 2.09 (1.56 - 2.52) | 1.19 - 3.22 | < 0.0001 |  |
| circ*CSNK1G3* | down | 1.56 (1.32 - 1.80) | 1.07 - 2.51 | < 0.0001 |  |
| circ*GUCY1A2* | – | 1.17 (0.96 - 1.41) | 0.71 - 2.01 | 0.067 |  |
| circ*LPP* | down | 1.64 (1.35 - 1.81) | 1.11 - 2.39 | < 0.0001 |  |
| circ*NEAT1* | up | 1.70 (1.13 - 3.02) | 0.85 - 4.57 | 0.003 |  |
| circ*RHOBTB3* | down | 1.49 (1.26 - 1.70) | 0.99 - 2.34 | < 0.0001 |  |
| circ*STIL* | down | 1.36 (1.07 - 1.58) | 0.91 - 1.89 | 0.0002 |  |
|  |  | *Linear transcripts* |  |  |  |
| lin*ATXN10* | – | 1.07 (0.99 - 1.12) | 0.90 - 1.27 | 0.133 |  |
| lin*CRIM1* | down | 1.50 (1.34 - 1.78) | 1.08 - 2.06 | < 0.0001 |  |
| lin*CSNK1G3* | – | 1.01 (0.94 - 1.07) | 0.85 - 1.18 | 0.841 |  |
| lin*GUCY1A2* | – | 1.12 (0.93 - 1.35) | 0.82 - 1.64 | 0.069 |  |
| lin*LPP* | down | 1.46 (1.32 - 1.76) | 1.06 - 2.23 | < 0.0001 |  |
| lin*NEAT1* | up | 1.75 (1.26 - 2.17) | 1.10 - 2.84 | < 0.0001 |  |
| lin*RHOBTB3* | – | 1.01 (0.93 - 1.05) | 0.80 - 1.16 | 0.558 |  |
| lin*STIL* | up | 1.88 (1.67 - 2.12) | 1.36 - 2.58 | < 0.0001 |  |

^a^ For *NEAT1*, only 45 paired samples could be compared. ^b^ Wilcoxon test of paired samples.

**Supplementary Table S9.** Expression of circRNAs and their linear transcripts in paired adjacent normal tissue samples and malignant tissue samples compared.

| **Circular RNA** | | **Linear Transcript** | | | ***p*-Value ^b^** |
| --- | --- | --- | --- | --- | --- |
| **Variable ^a^** | **Median (95% CI)** | | **Variable ^a^** | **Median (95% CI)** |  |
| circ*ATXN10* ^N^ | 0.392 (0.345–0.429) | | lin*ATXN10* ^N^ | 4.09 (3.95-4.27) | < 0.0001 |
| circ*ATXN10* ^T^ | 0.200 (0.170–0.230) | | lin*ATXN10* ^T^ | 3.81 (3.71–4.16) | < 0.0001 |
| circ*CRIM1* ^N^ | 0.496 (0.412–0.551) | | lin*CRIM1* ^N^ | 3.86 (3.63–4.10) | < 0.0001 |
| circ*CRIM1* ^T^ | 0.243 (0.189–0.305) | | lin*CRIM1* ^T^ | 2.54 (2.19–2.81) | < 0.0001 |
| circ*CSNK1G3* ^N^ | 1.53 (1.42–1.81) | | lin*CSNK1G3* ^N^ | 0.878 (0.825–0.941) | < 0.0001 |
| circ*CSNK1G3* ^T^ | 0.925 (0.820–1.12) | | lin*CSNK1G3* ^T^ | 0.898 (0.798–0.971) | 0.0262 |
| circ*GUCY1A2* ^N^ | 0.719 (0.625–0.887) | | lin*GUCY1A2* ^N^ | 1.68 (1.48–2.06) | < 0.0001 |
| circ*GUCY1A2* ^T^ | 0.605 (0.502–0.711) | | lin*GUCY1A2* ^T^ | 1.41 (1.30–1.52) | < 0.0001 |
| circ*LPP* ^N^ | 0.146 (0.132–0.168) | | lin*LPP* ^N^ | 12.5 (11.6–13.5) | < 0.0001 |
| circ*LPP* ^T^ | 0.085 (0.077–0.102) | | lin*LPP* ^T^ | 7.84 (6.71–8.93) | < 0.0001 |
| circ*NEAT1* ^N^ | 1.90x10^-3^ (1.49x10^-3^–2.45x 10^-3^ ) | | lin*NEAT1* ^N^ | 317 (297–348) | < 0.0001 |
| circ*NEAT1* ^T^ | 3.20x10^-3^ (2.23x10^-3^–4.15x10^-3^) | | lin*NEAT1* ^T^ | 491 (410–762) | < 0.0001 |
| circ*RHOBTB3* ^N^ | 0.399 (0.339–0.434) | | lin*RHOBTB3* ^N^ | 3.68 (3.54–3.90) | < 0.0001 |
| circ*RHOBTB3* ^T^ | 0.260 (0.227–0.304 | | lin*RHOBTB3* ^T^ | 3.69 (3.39–4.06) | < 0.0001 |
| circ*STIL* ^N^ | 0.391 (0.360–0.428) | | lin*STIL* ^N^ | 2.07 (1.91–2.17) | < 0.0001 |
| circ*STIL* ^T^ | 0.300 (0.286–0.333) | | lin*STIL* ^T^ | 3.94 (3.66–4.48) | < 0.0001 |

^a^ Superscripts of the variables indicate sample sources: N, adjacent normal tissue; T, malignant tissue. ^b^ Wilcoxon test for paired samples.

**Supplementary Table S10.** Expression ratios of linear to circular RNAs in paired adjacent normal tissue and malignant tissue samples. ^a^

| **Ratio** | **Ratio of Linear to Circular RNA in Normal and Tumor Tissue** | | |
| --- | --- | --- | --- |
|  | **Median (95% CI)** | | ***p-*Value ^b^** |
|  | *Adjacent normal tissue* | *Tumor tissue* |  |
| lin*ATXN10*/circ*ATXN10* | 10.8 (9.16 - 11.8) | 19.4 (17.4 - 23.4) | < 0.0001 |
| lin*CRIM1*/circ*CRIM1* | 8.28 (7.81 - 9.12) | 10.7 (9.87 - 12.3) | < 0.0001 |
| lin*CSNK1G3*/circ*CSNK1G3* | 0.55 (0.51 - 0.61) | 0.95 (0.82 - 1.07) | < 0.0001 |
| lin*GUCY1A2*/circ*GUCY1A2* | 2.31 (2.06 - 2.65) | 2.38 (2.11 - 2.74) | 0.467 |
| lin*LPP*/circ*LPP* | 82.4 (81.0 - 84.3) | 88.6 (85.6 - 90.9) | < 0.0001 |
| lin*NEAT1*/circ*NEAT1* | 17.4x10^3^ (12.1x10^3^ - 24.6x10^3^) | 17.9x10^3^ (14.7x10^3^ - 23.1x10^3^) | 0.906 |
| lin*RHOBTB3*/circ*RHOBTB3* | 9.88 (8.66 - 11.8) | 12.5 (10.6 - 15.9) | < 0.0001 |
| lin*STIL*/circ*STIL* | 4.79 (4.34 - 5.71) | 13.4 (11.9 - 15.2) | < 0.0001 |

^a^ For *NEAT1*, only 45 paired samples could be compared. ^b^ Wilcoxon test of paired samples.

**Supplementary Table S11.** Associations of circRNAs and linear transcripts with clinicopathological variables. The associations were calculated by Mann-Whitney U-test. The continuous data (age, preoperative PSA, and prostate volume) were dichotomized according to the medians listed in Table 1 and the categorized TNM stages and ISUP grades were classified according to pT1+2 to pT3 and ISUP grades 1–3 to ISUP 4+5, respectively. Details of the 115 patients and the abbreviations used are given in Table 1 of the Main text. *P*-values of the results are indicated; statistically significant associations were highlighted in yellow. For circ*NEAT1*, only 86 sample data could be included in the association calculations (see Supplementary Table S7).

**a) Circular RNAs**

| **Variables** | **circ**  ***ATXN10*** | **circ**  ***CRIM1*** | **circ**  ***CSNK1G3*** | **circ**  ***GUCY1A2*** | **circ**  ***LPP*** | **circ**  ***NEAT1*** | **circ**  ***RHOBTB3*** | **circ**  ***STIL*** |
| --- | --- | --- | --- | --- | --- | --- | --- | --- |
| Age | *p*=0.677 | *p*=0.633 | *p*=0.949 | *p*=0.226 | *p*=0.811 | *p*=0.249 | *p*=0.140 | *p*=0.803 |
| PSA | *p*=0.681 | *p*=0.761 | *p*=0.346 | *p*=0.615 | *p*=0.735 | *p*=0.832 | *p*=0.812 | *p*=0.631 |
| Prostate volume | *p*=0.656 | *p*=0.862 | *p*=0.978 | *p*=0.796 | *p*=0.497 | *p*=0.232 | *p*=0.337 | *p*=0.146 |
| DRE | *p*=0.138 | *p*=0.056 | *p*=0.234 | *p*=0.967 | *p*=0.169 | *p*=0.184 | *p*=0.187 | *p*=0.055 |
| TNM | *p*=0.106 | *p*=0.483 | *p*=0.551 | *p*=0.729 | *p*=0.398 | *p*=0.636 | *p*=0.594 | *p*=0.108 |
| ISUP | *p*=0.037 | *p*=0.024 | *p*=0.041 | *p*=0.010 | *p*=0.010 | *p*=0.334 | *p*=0.945 | *p*=0.048 |
| R status | *p*=0.924 | *p*=0.384 | *p*=0.296 | *p*=0.839 | *p*=0.195 | *p*=0.692 | *p*=0.913 | *p*=0.059 |

**b) Linear Transcripts**

| **Variables** | **lin**  ***ATXN10*** | **lin**  ***CRIM1*** | **lin**  ***CSNK1G3*** | **lin**  ***GUCY1A2*** | **lin**  ***LPP*** | **lin**  ***NEAT1*** | **lin**  ***RHOBTB3*** | **lin**  ***STIL*** |
| --- | --- | --- | --- | --- | --- | --- | --- | --- |
| Age | *p*=0.208 | *p*=0.270 | *p*=0.296 | *p*=0.498 | *p*=0.509 | *p*=0.106 | *p*=0.667 | *p*=0.458 |
| PSA | *p*=0.064 | *p*=0.685 | *p*=0.294 | *p*=0.814 | *p*=0.623 | *p*=0.337 | *p*=0.942 | *p*=0.452 |
| Prostate volume | *p*=0.302 | *p*=0.408 | *p*=0.121 | *p*=0.711 | *p*=0.788 | *p*=0.829 | *p*=0.337 | *p*=0.922 |
| DRE | *p*=0.369 | *p*=0.733 | *p*=0.595 | *p*=0.937 | *p*=0.137 | *p*=0.160 | *p*=0.073 | *p*=0.540 |
| TNM | *p*=0.179 | *p*=0.226 | *p*=0.716 | *p*=0.235 | *p*=0.314 | *p*=0.128 | *p*=0.014 | *p*=0.149 |
| ISU*P* | *p*=0.777 | *p*=0.094 | *p*=0.934 | *p*=0.028 | *p*=0.007 | *p*=0.450 | *p*=0.229 | *p*=0.238 |
| R status | *p*=0.951 | *p*=0.093 | *p*=0.170 | *p*=0.703 | *p*=0.182 | *p*=0.978 | *p*=0.357 | *p*=0.172 |

**Supplementary Table S12.** Spearman rank correlation coefficients between all circRNAs in adjacent normal tissue samples from prostate cancer specimens (paired samples, n = 79; only 45 for circ*NEAT1*). Statistically significant correlation coefficients (*p* < 0.05) are indicated by yellow-highlighted table fields.

| **RNA** | r_s_ | **circ**  ***ATXN10*** | **circ**  ***CRIM1*** | **circ**  ***CSNK1G3*** | **circ**  ***GUCY1A2*** | **circ**  ***LPP*** | **circ**  ***NEAT1*** | **circ**  ***RHOBTB3*** | **circ**  ***STIL*** |
| --- | --- | --- | --- | --- | --- | --- | --- | --- | --- |
| circ  *ATXN10* | r_s_  *p-*value |  | 0.764 <0.0001 | 0.803 <0.0001 | 0.284 0.0112 | 0.698 <0.0001 | 0.190 0.1524 | 0.390 0.0004 | 0.724 <0.0001 |
| circ  *CRIM1* | r_s_  *p-*value | 0.764 <0.0001 |  | 0.741 <0.0001 | 0.315 0.0046 | 0.634 <0.0001 | 0.064 0.6321 | 0.007 0.9517 | 0.577 <0.0001 |
| circ  *CSNK1G3* | r_s_  *p-*value | 0.803 <0.0001 | 0.741 <0.0001 |  | 0.335 0.0025 | 0.682 <0.0001 | 0.117 0.3808 | 0.256 0.0226 | 0.773 <0.0001 |
| circ  *GUCY1A2* | r_s_  *p-*value | 0.284 0.0112 | 0.315 0.0046 | 0.335 0.0025 |  | 0.331 0.0029 | -0.100 0.4571 | 0.049 0.6650 | 0.136 0.2308 |
| circ  *LPP* | r_s_  *p-*value | 0.698 <0.0001 | 0.634 <0.0001 | 0.682 <0.0001 | 0.331 0.0029 |  | 0.263 0.0465 | 0.509 <0.0001 | 0.558 <0.0001 |
| circ  *NEAT1* | r_s_  *p-*value | 0.190 0.1524 | 0.064 0.6321 | 0.117 0.3808 | -0.100 0.4571 | 0.263 0.0465 |  | 0.192 0.1487 | 0.082 0.5404 |
| circ  *RHOBTB3* | r_s_  *p-*value | 0.390 0.0004 | 0.007 0.9517 | 0.256 0.0226 | 0.049 0.6650 | 0.509 <0.0001 | 0.192 0.1487 |  | 0.194 0.0867 |
| circ  *STIL* | r_s_  *p-*value | 0.724 <0.0001 | 0.577 <0.0001 | 0.773 <0.0001 | 0.136 0.2308 | 0.558 <0.0001 | 0.082 0.5404 | 0.194 0.0867 |  |

**Supplementary Table S13.** Spearman rank correlation coefficients between all circRNAs in malignant tissue samples from prostate cancer specimens (paired samples, n=79; only 45 for circ*NEAT1*). Statistically significant correlation coefficients (*p* < 0.05) are indicated by yellow-highlighted table fields.

| **RNA** | r_s_ | **circ**  ***ATXN10*** | **circ**  ***CRIM1*** | **circ**  ***CSNK1G3*** | **circ**  ***GUCY1A2*** | **circ**  ***LPP*** | **circ**  ***NEAT1*** | **circ**  ***RHOBTB3*** | **circ**  ***STIL*** |
| --- | --- | --- | --- | --- | --- | --- | --- | --- | --- |
| circ  *ATXN10* | r_s_  *p*-value |  | 0.827 <0.0001 | 0.785 <0.0001 | 0.489 <0.0001 | 0.814 <0.0001 | 0.003 0.9803 | 0.446 <0.0001 | 0.665 <0.0001 |
| circ  *CRIM1* | r_s_  *p*-value | 0.827 <0.0001 |  | 0.707 <0.0001 | 0.575 <0.0001 | 0.771 <0.0001 | 0.014 0.9139 | 0.181 0.1108 | 0.559 <0.0001 |
| circ  *CSNK1G3* | r_s_  *p*-value | 0.785 <0.0001 | 0.707 <0.0001 |  | 0.458 <0.0001 | 0.801 <0.0001 | 0.126 0.3361 | 0.292 0.0090 | 0.746 <0.0001 |
| circ  *GUCY1A2* | r_s_  *p*-value | 0.489 <0.0001 | 0.575 <0.0001 | 0.458 <0.0001 |  | 0.509 <0.0001 | 0.111 0.3966 | -0.039 0.7348 | 0.349 0.0016 |
| circ  *LPP* | r_s_  *p*-value | 0.814 <0.0001 | 0.771 <0.0001 | 0.801 <0.0001 | 0.509 <0.0001 |  | 0.075 0.5706 | 0.385 0.0005 | 0.540 <0.0001 |
| circ  *NEAT1* | r_s_  *p*-value | 0.003 0.9803 | 0.014 0.9139 | 0.126 0.3361 | 0.111 0.3966 | 0.075 0.5706 |  | -0.045 0.7353 | -0.023 0.8610 |
| circ  *RHOBTB3* | r_s_  *p*-value | 0.446 <0.0001 | 0.181 0.1108 | 0.292 0.0090 | -0.039 0.7348 | 0.385 0.0005 | -0.045 0.7353 |  | 0.137 0.2300 |
| circ  *STIL* | r_s_  *p*-value | 0.665 <0.0001 | 0.559 <0.0001 | 0.746 <0.0001 | 0.349 0.0016 | 0.540 <0.0001 | -0.023 0.8610 | 0.137 0.2300 |  |

**Supplementary Table S14.** Spearman rank correlation coefficients between circRNAs in paired adjacent normal tissue samples and malignant tissue samples from prostate cancer specimens (n=79, only 45 for circ*NEAT1*). Statistically significant correlation coefficients (*p* <0.05) are indicated by yellow-highlighted table fields.

| **circRNAs in Tumor** | **r_s_** | **circRNAs in Adjacent Normal Tissue Samples** | | | | | | | |
| --- | --- | --- | --- | --- | --- | --- | --- | --- | --- |
|  |  | **circ**  ***ATXN10*** | **circ**  ***CRIM1*** | **circ**  ***CSNK1G3*** | **circ**  ***GUCY1A2*** | **circ**  ***LPP*** | **circ**  ***NEAT1*** | **circ**  ***RHOBTB3*** | **circ**  ***STIL*** |
| circ  *ATXN10* | r_s_  *p*-value | 0.136 0.2328 | 0.048 0.6765 | 0.050 0.6597 | 0.240 0.0334 | 0.186 0.1003 | 0.188 0.1576 | 0.016 0.8853 | 0.194 0.0864 |
| circ  *CRIM1* | r_s_  *p*-value | 0.137 0.2288 | 0.216 0.0564 | 0.109 0.3386 | 0.160 0.1580 | 0.168 0.1388 | 0.108 0.4205 | -0.154 0.1740 | 0.238 0.0343 |
| circ  *CSNK1G3* | r_s_  *p*-value | 0.157 0.1682 | 0.162 0.1546 | 0.251 0.0254 | 0.267 0.0173 | 0.309 0.0056 | 0.147 0.2701 | -0.012 0.9142 | 0.305 0.0063 |
| circ  *GUCY1A2* | r_s_  *p*-value | 0.134 0.2382 | 0.167 0.1415 | 0.129 0.2555 | 0.380 0.0005 | 0.110 0.3362 | -0.037 0.7818 | -0.171 0.1319 | 0.030 0.7915 |
| circ  *LPP* | r_s_  *p*-value | 0.121 0.2895 | 0.091 0.4276 | 0.115 0.3149 | 0.192 0.0896 | 0.307 0.0059 | 0.208 0.1173 | 0.014 0.9033 | 0.221 0.0507 |
| circ  *NEAT1* | r_s_  *p*-value | 0.036 0.7864 | -0.005 0.9686 | 0.114 0.3868 | -0.088 0.5013 | -0.077 0.5611 | 0.066 0.6644 | -0.037 0.7762 | 0.024 0.8564 |
| circ  *RHOBTB3* | r_s_  *p*-value | -0.024 0.8367 | -0.318 0.0043 | -0.088 0.4389 | 0.227 0.0439 | 0.126 0.2673 | 0.183 0.1696 | 0.473 <0.0001 | -0.118 0.3005 |
| circ  *STIL* | r_s_  *p*-value | 0.165 0.1450 | 0.065 0.5686 | 0.110 0.3351 | 0.131 0.2508 | 0.180 0.1127 | -0.076 0.5725 | -0.062 0.5849 | 0.279 0.0129 |

**Supplementary Table S15.** Spearman rank correlation coefficients between circRNAs and their linear counterparts in paired adjacent normal tissue and tumor samples from prostate cancer specimens (n=79, only 45 for circ*NEAT1*). Statistically significant differences between the correlation coefficients are yellow-highlighted.

|  | **Correlation Coefficient r_S_ (*p*-Value)** | |  |
| --- | --- | --- | --- |
| **Correlation Pairs** | **Adjacent Normal Tissue** | **Tumor Tissue** | ***p*-Value (Difference Between the r_S_ )** |
| circ*ATXN10*  with lin*ATXN10* | 0.003 (0.9779) | 0.312 (0.0051) | 0.0487 |
| circ*CRIM1*  with lin*CRIM1* | 0.715 (< 0.0001) | 0.776 (< 0.0001) | 0.3953 |
| circ*CSNK1G3*  with lin*CSNK1G3* | 0.387 (0.0004) | 0.402 (0.0002) | 0.9128 |
| circ*GUCY1A2*  with lin*GUCY1A2* | 0.709 (< 0.0001) | 0.728 (< 0.0001) | 0.8086 |
| circ*LPP*  with lin*LPP* | 0.943 (< 0.0001) | 0.950 (< 0.0001) | 0.6782 |
| circ*NEAT1*  with lin*NEAT1* | 0.240 (0.0695) | 0.582 (< 0.0001) | 0.0095 |
| circ*RHOBTB3*  with lin*RHOBTB3* | 0.229 (0.0422) | 0.437 (0.0001) | 0.1468 |
| circ*STIL*  with lin*STIL* | -0.270 (0.0163) | 0.207 (0.0667) | 0.0063 |

**Supplementary Information S6: Cox regression analyses, C-statistics data, and decision curve analyses of BCR prediction models**

**Supplementary Table S16.** C-statistics of prognostic indices of the two RNA-based BCR prediction models regarding their predictive ability of biochemical recurrence.

| **Prediction Models ^a^** | **C-Statistics**  **Mean ± SE** | ***p*-Value** | |
| --- | --- | --- | --- |
|  |  | **Within**  **Models** | c**ircRNA-Based vs. linRNA-Based Mode**l |
| circRNA-based prediction model |  |  | 0.141 |
| Full model  Reduced model | 0.676 ± 0.0550  0.649 ± 0.0559 | 0.219 |  |
| linRNA-based prediction model |  |  |  |
| Full model + Reduced model | 0.722 ± 0.0528 |  |  |

Abbreviations: BCR, biochemical recurrence; SE, standard error. ^a^ The prediction models refer to the description of the "full" and "reduced" models in Table 6 of the Main text.

**Supplementary Table S17.** Univariate Cox regression data of 8 linear RNAs as BCR predictors in this study using The Cancer Genome Atlas Prostate Cancer (TCGA-PRAD) data set.

|  | **Univariate Cox Regression ^a^** | | |  |
| --- | --- | --- | --- | --- |
| **linRNA** | **Concordance**  **(Mean ± SE)** | **Wald statistics** | **HR (95% CI)** | ***p‑*Value** |
| lin*ATXN10* | 0.522 ± 0.038 | 0.291 | 1.000 (0.9997–1.000) | 0.590 |
| lin*CRIM1* | 0.540 ± 0.034 | 0.901 | 0.9999(0.9999–1.000) | 0.343 |
| lin*CSNK1G3* | 0.512 ± 0.036 | 0.249 | 1.000 (0.9991–1.001) | 0.618 |
| lin*GUCY1A2* | 0.487 ± 0.034 | 0.428 | 1.003 (0.9941–1.012) | 0.513 |
| lin*LPP* | 0.503 ± 0.036 | 0.075 | 0.9999 (0.9995–1.000) | 0.784 |
| lin*NEAT1* | 0.530 ± 0.032 | 0.384 | 1.000 (1.000–1.000) | 0.535 |
| lin*RHOBTB3* | 0.615 ± 0.035 | 3.401 | 0.9997 (0.9994–1.000) | 0.065 |
| lin*STIL* | 0.595 ± 0.037 | 17.58 | 1.003 (1.002–1.005) | 0.00003 |

Abbreviations: BCR, biochemical recurrence; CI, confidence interval; Concordance, C-index according to Harrell et al. [19]; HR, hazard ratio; SE, standard error. ^a^ TCGA-PRAD RNAseq and clinical data were downloaded and analyzed in R (version 3.6.3) using the "TCGA2stat" library and the "survival" library. Univariate Cox regression analysis was performed for each individual linear transcript.


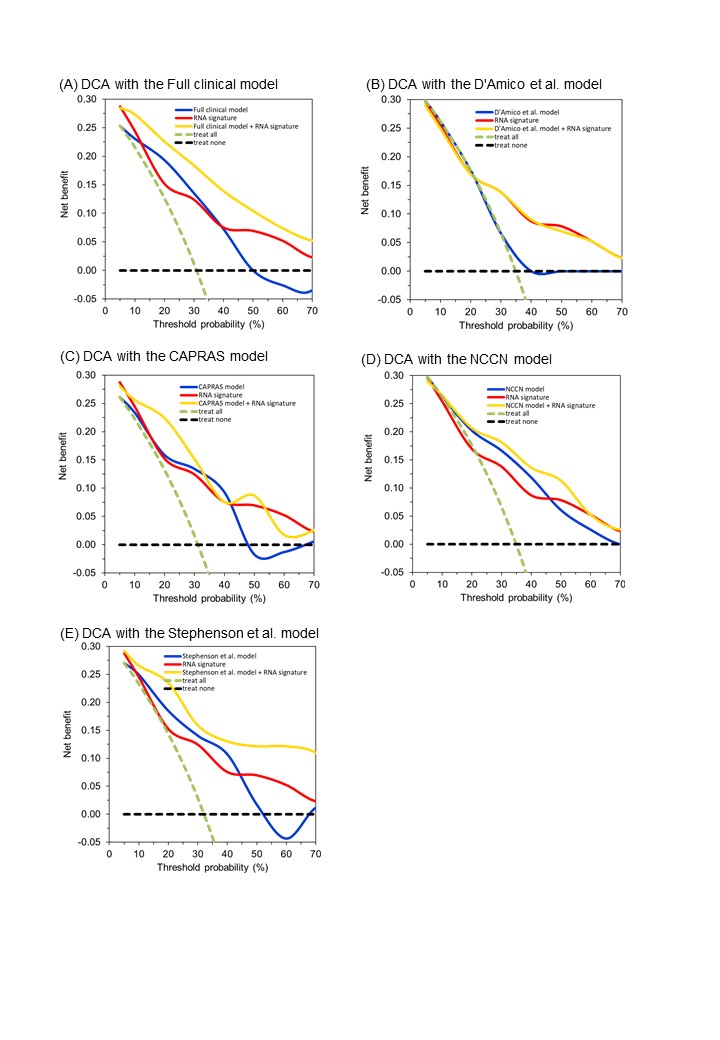


**Supplementary Figure S4.** Decision curve analysis (DCA) of clinicopathological-based prediction tools of biochemical recurrence to demonstrate the predictive benefit by the inclusion of the developed RNA signature. Data refer to the models described in Table 8 of the Main text, according to (**A**) the "full clinical model" established in the present study, (**B**) D'Amico et al. [20], (**C**) Cancer of the Prostate Risk Assessment Postsurgical Score [21], (**D**) National Comprehensive Cancer Network [22], and Stephenson et al. [23].

**References**

1. Glazar, P.; Papavasileiou, P.; Rajewsky, N. circBase: a database for circular RNAs. *RNA* **2014,** *20*, 1666-1670.

2. Dudekula, D. B.; Panda, A. C.; Grammatikakis, I.; De, S.; Abdelmohsen, K.; Gorospe, M. CircInteractome: a web tool for exploring circular RNAs and their interacting proteins and microRNAs. *RNA Biol.* **2016,** *13*, 34-42.

3. Wong, N.; Wang, X. miRDB: an online resource for microRNA target prediction and functional annotations. *Nucleic Acids Res.* **2015,** *43*, D146-D152.

4. Agarwal, V.; Bell, G. W.; Nam, J. W.; Bartel, D. P. Predicting effective microRNA target sites in mammalian mRNAs. *Elife* **2015,** *4*.

5. Bustin, S. A.; Benes, V.; Garson, J. A.; Hellemans, J.; Huggett, J.; Kubista, M.; Mueller, R.; Nolan, T.; Pfaffl, M. W.; Shipley, G. L.; et al. The MIQE guidelines: minimum information for publication of quantitative real-time PCR experiments. *Clin. Chem.* **2009,** *55*, 611-622.

6. Franz, A.; Ralla, B.; Weickmann, S.; Jung, M.; Rochow, H.; Stephan, C.; Erbersdobler, A.; Kilic, E.; Fendler, A.; Jung, K. Circular RNAs in clear cell renal cell carcinoma: their microarray-based identification, analytical validation, and potential use in a clinico-genomic model to improve prognostic accuracy. *Cancers (Basel)* **2019,** *11*, E1473.

7. Ohl, F.; Jung, M.; Xu, C.; Stephan, C.; Rabien, A.; Burkhardt, M.; Nitsche, A.; Kristiansen, G.; Loening, S. A.; Radonic, A.; et al. Gene expression studies in prostate cancer tissue: which reference gene should be selected for normalization? *J. Mol. Med.* **2005,** *83*, 1014-1024.

8. Chen, S.; Huang, V.; Xu, X.; Livingstone, J.; Soares, F.; Jeon, J.; Zeng, Y.; Hua, J. T.; Petricca, J.; Guo, H.; et al. Widespread and functional RNA circularization in localized prostate cancer. *Cell* **2019,** *176*, 831-843.

9. Zhang, C.; Xiong, J.; Yang, Q.; Wang, Y.; Shi, H.; Tian, Q.; Huang, H.; Kong, D.; Lv, J.; Liu, D.; et al. Profiling and bioinformatics analyses of differential circular RNA expression in prostate cancer cells. *Future. Sci. OA.* **2018,** *4*, FSOA340.

10. Jeck, W. R.; Sharpless, N. E. Detecting and characterizing circular RNAs. *Nat. Biotechnol.* **2014,** *32*, 453-461.

11. Untergasser, A.; Cutcutache, I.; Koressaar, T.; Ye, J.; Faircloth, B. C.; Remm, M.; Rozen, S. G. Primer3 - new capabilities and interfaces. *Nucleic Acids Res.* **2012,** *40*, e115.

12. Salzman, J.; Chen, R. E.; Olsen, M. N.; Wang, P. L.; Brown, P. O. Cell-type specific features of circular RNA expression. *PLoS Genet.* **2013,** *9*, e1003777.

13. Szabo, L.; Salzman, J. Detecting circular RNAs: bioinformatic and experimental challenges. *Nat. Rev. Genet.* **2016,** *17*, 679-692.

14. Memczak, S.; Jens, M.; Elefsinioti, A.; Torti, F.; Krueger, J.; Rybak, A.; Maier, L.; Mackowiak, S. D.; Gregersen, L. H.; Munschauer, M.; et al. Circular RNAs are a large class of animal RNAs with regulatory potency. *Nature* **2013,** *495*, 333-338.

15. Lu, T.; Cui, L.; Zhou, Y.; Zhu, C.; Fan, D.; Gong, H.; Zhao, Q.; Zhou, C.; Zhao, Y.; Lu, D.; et al. Transcriptome-wide investigation of circular RNAs in rice. *RNA* **2015,** *21*, 2076-2087.

16. Westholm, J. O.; Miura, P.; Olson, S.; Shenker, S.; Joseph, B.; Sanfilippo, P.; Celniker, S. E.; Graveley, B. R.; Lai, E. C. Genome-wide analysis of drosophila circular RNAs reveals their structural and sequence properties and age-dependent neural accumulation. *Cell Rep.* **2014,** *9*, 1966-1980.

17. Zhong, S.; Zhou, S.; Yang, S.; Yu, X.; Xu, H.; Wang, J.; Zhang, Q.; Lv, M.; Feng, J. Identification of internal control genes for circular RNAs. *Biotechnol. Lett.* **2019,** *41*, 1111-1119.

18. Jeck, W. R.; Sorrentino, J. A.; Wang, K.; Slevin, M. K.; Burd, C. E.; Liu, J.; Marzluff, W. F.; Sharpless, N. E. Circular RNAs are abundant, conserved, and associated with ALU repeats. *RNA* **2013,** *19*, 141-157.

19. Harrell Jr, F. E.; Lee, K. L.; Mark, D. B. Multivariable prognostic models: issues in developing models, evaluating assumptions and adequacy, and measuring and reducing errorrs. *Stat. Med.* **1996,** *15*, 361-387.

20. D'Amico, A. V.; Whittington, R.; Malkowicz, S. B.; Schultz, D.; Blank, K.; Broderick, G. A.; Tomaszewski, J. E.; Renshaw, A. A.; Kaplan, I.; Beard, C. J.; et al. Biochemical outcome after radical prostatectomy, external beam radiation therapy, or interstitial radiation therapy for clinically localized prostate cancer. *JAMA* **1998,** *280*, 969-974.

21. Cooperberg, M. R.; Pasta, D. J.; Elkin, E. P.; Litwin, M. S.; Latini, D. M.; Du Chane, J.; Carroll, P. R. The University of California, San Francisco Cancer of the Prostate Risk Assessment score: a straightforward and reliable preoperative predictor of disease recurrence after radical prostatectomy. *J. Urol.* **2005,** *173*, 1938-1942.

22. Mohler, J. L. The 2010 NCCN clinical practice guidelines in oncology on prostate cancer. *J. Natl. Compr. Canc. Netw.* **2010,** *8*, 145.

23. Stephenson, A. J.; Scardino, P. T.; Eastham, J. A.; Bianco, F. J., Jr.; Dotan, Z. A.; DiBlasio, C. J.; Reuther, A.; Klein, E. A.; Kattan, M. W. Postoperative nomogram predicting the 10-year probability of prostate cancer recurrence after radical prostatectomy. *J. Clin. Oncol.* **2005,** *23*, 7005-7012.
